# Automatic Morphological Subtyping Reveals New Roles of Caspases in Mitochondrial Dynamics

**DOI:** 10.1371/journal.pcbi.1002212

**Published:** 2011-10-06

**Authors:** Jyh-Ying Peng, Chung-Chih Lin, Yen-Jen Chen, Lung-Sen Kao, Young-Chau Liu, Chung-Chien Chou, Yi-Hung Huang, Fang-Rong Chang, Yang-Chang Wu, Yuh-Show Tsai, Chun-Nan Hsu

**Affiliations:** 1 Institute of Biomedical Informatics, National Yang-Ming University, Taipei, Taiwan; 2 Taipei City Hospital, Taipei, Taiwan; 3 Department of Life Sciences and Institute of Genome Sciences, National Yang-Ming University, Taipei, Taiwan; 4 Institute of Information Science, Academia Sinica, Taipei, Taiwan; 5 College of Liberal Education, Shu-Te University, Kaohsiung City, Taiwan; 6 Department of Computer Science and Information Engineering, National Taiwan University, Taipei, Taiwan; 7 Graduate Institute of Natural Products, Kaohsiung Medical University, Kaohsiung, Taiwan; 8 Graduate Institute of Integrated Medicine, China Medical University, Taichung, Taiwan; 9 Graduate Institute of Biomedical Engineering, Chung-Yuan Christian University, Zhongli City, Taiwan; 10 Information Sciences Institute, University of Southern California, Marina del Rey, California, United States of America; Stanford University, United States of America

## Abstract

Morphological dynamics of mitochondria is associated with key cellular processes related to aging and neuronal degenerative diseases, but the lack of standard quantification of mitochondrial morphology impedes systematic investigation. This paper presents an automated system for the quantification and classification of mitochondrial morphology. We discovered six morphological subtypes of mitochondria for objective quantification of mitochondrial morphology. These six subtypes are small globules, swollen globules, straight tubules, twisted tubules, branched tubules and loops. The subtyping was derived by applying consensus clustering to a huge collection of more than 200 thousand mitochondrial images extracted from 1422 micrographs of Chinese hamster ovary (CHO) cells treated with different drugs, and was validated by evidence of functional similarity reported in the literature. Quantitative statistics of subtype compositions in cells is useful for correlating drug response and mitochondrial dynamics. Combining the quantitative results with our biochemical studies about the effects of squamocin on CHO cells reveals new roles of Caspases in the regulatory mechanisms of mitochondrial dynamics. This system is not only of value to the mitochondrial field, but also applicable to the investigation of other subcellular organelle morphology.

## Author Summary

Mitochondria are “cellular power plants” that synthesize adenosine triphosphate (ATP) from degradation of nutrients, providing chemical energy for cellular activities. In addition, mitochondria are involved in a range of other cellular processes, such as signaling, cell differentiation, cell death, cell cycle and cell growth. Dysfunctional mitochondrial dynamics have been linked to several neurodegenerative diseases, and may play a role in the aging process. Previous studies on the correlation between mitochondrial morphological changes and pathological processes involve mostly manual or semi-automated classification and quantification of morphological features, which introduces biases and inconsistency, and are labor intensive. In this work we have developed an automated quantification system for mitochondrial morphology, which is able to extract and distinguish six representative morphological subtypes within cells. Using this system, we have analyzed 1422 cells and extracted more than 200 thousand individual mitochondrion, and calculated morphological statistics for each cell. From the numerical results we were able to derive new biological conclusions about mitochondrial morphological dynamics. With this new system, investigations of mitochondrial morphology can be scaled up and objectively quantified, allowing standardization of morphological distinctions and replicability between experiments. This system will facilitate future research on the relation between subcellular morphology and various physiological processes.

## Introduction

Recent studies have shown that the fusion-fission dynamics of mitochondria are essential to many cellular processes, including ATP-level maintenance, redox signaling, oxidative stress generation, and cell death [1]–[4]. Meanwhile, it is also known that dysfunctional mitochondrial dynamics ushers the aging process and neuronal degenerative diseases [5]–[11]. Since mitochondrial morphology reveals physiological and pathological status, tracking mitochondrial morphological differences becomes important. Previous studies roughly classified mitochondrial morphology into various subtypes, such as, fragmented globules, tubular threads, networks, clumps or swollen granules, and usually the classification was performed by human inspection [2], [8], [9], [12], which inevitably introduces biases and inconsistency and lowers replicability of the results. Previous attempts of automatic quantification include measuring length, width, area and other primitive parameters of mitochondrial objects [13] and also skeleton length [14], but these measures are insufficient to fully distinguish the morphological diversity of mitochondria. They [13], [14] investigated only the average of these feature values within each cell, while in this paper, we present a computational approach that allows us to identify representative morphological subtypes and quantify the morphological diversity of mitochondria.

Our approach consists of successive steps of image segmentation, consensus clustering and classifier learning designed to identify subtypes as well as construct a subtype classifier. A large set of fluorescent microscopic images of Chinese Hamster Ovary (CHO) cells were used as the sample to derive the subtypes. A subset of these CHO cells was treated with squamocin, a compound known to induce apoptosis [15]–[18]. Squamocin treatment results in mitochondrial fragmentation, which can then be suppressed by inhibitors of Caspases 8 and 9 (z-IETD and z-LEHD, respectively), but cells are still killed by squamocin even with the presence of these inhibitors [19]. One possible reason is that Caspase inhibitors may not have fully restored mitochondrial structures. With the developed computational approach, we were able to quantify the difference of morphological changes of mitochondria in cells under different treatments.

We used a previously developed image segmentation method [20] to accurately extract each individual mitochondrial object from cell micrographs. This method applies adaptive local normalization [20] and Otsu's image thresholding method [21] to deal with noisy background and variant object intensity that are constantly present in fluorescent micrographs, a challenging issue for existing image binarization methods. We then applied this method to extract a large number of mitochondrial objects. Each object was then represented by a set of image features, including morphological, skeletal, and binary Haralick texture features. According to result of consensus clustering of the image features, we merged clusters that are functionally similar to define six morphological subtypes of mitochondria. This new subtyping covers mitochondrial morphology reported in the literature and provides an unambiguous observable indicator of the status of a mitochondrion in a cell.

We then applied supervised learning algorithms to train an automatic classification system to classify mitochondrial objects in images into one of the six subtypes. The resulting system combined with object extraction was named “MicroP,” which allows us to accurately measure mitochondrial subtype compositions in individual cells to profile the outcome of different drug treatments.

We used MicroP to study the effect of squamocin, a compound known to induce cell apoptosis by triggering mitochondrial fragmentation. Inhibitors of Caspases 8 and 9 can partially rescue the fragmentation but the mechanism was not previously characterized. Analyzing subtype composition of cells treated by squamocin and Caspase inhibitors reveals a more comprehensive picture of how Caspases 8 and 9 interact with mitochondrial fusion-fission regulatory proteins to influence morphology and functions of mitochondria.

## Results

### Morphological subtyping of mitochondria

**Figure 1:**
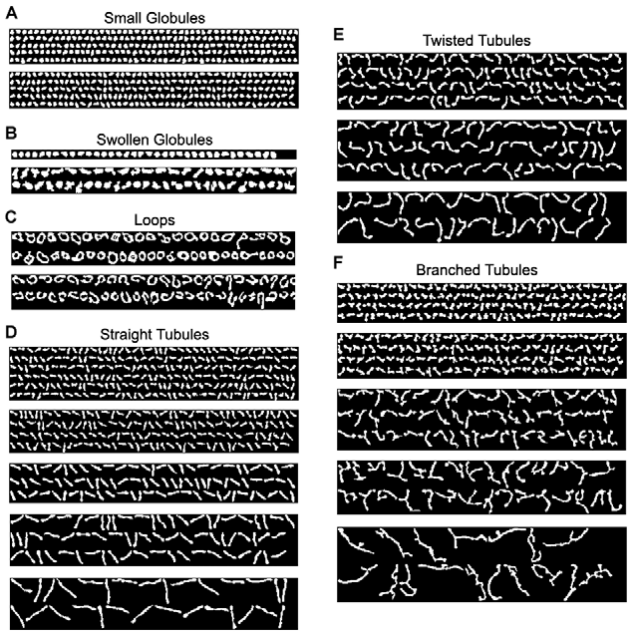
Representative clusters and subtyping reveal the range of morphological diversity of mitochondria in CHO cells. (A) Small globules: small round fragmented globules. (B) Swollen globules: large round and irregular-shaped globules. (C) Loops: Donuts and horseshoes. (D) Straight tubules: linear tubules with different lengths. (E) Twisted tubules: curved tubules with different lengths. (F) Branched tubules: branched tubules with different lengths.

We extracted 225,556 mitochondrial objects from the whole dataset of 1422 cells, and a total of 19 distinct morphologies were identified by consensus clustering with manual validation as shown in Figure 1. Inspecting these morphologies reveals that these automatically derived morphological clusters indeed have distinctive shapes.

To develop representative morphological subtypes for automatic classification, we further regrouped these 19 clusters according to morphological similarity and molecular functional characteristics, supported by evidence reported in the literature. The result is six subtypes: small globules, swollen globules, straight tubules, twisted tubules, branched tubules and loops. These subtypes provide useful indicators of specific cell conditions, and will be used as a basis for generating manually labeled mitochondria as training sets.

Small globules (Figure 1A) comprise the largest clusters in the clustering results. Swollen globules (Figure 1B) are in fact found as outliers in the clusters of small globules, since their number is too small to be distinguished by unsupervised clustering. But they are an important morphological subtype of mitochondria, suggested to represent dysfunctional mitochondria undergoing mitophagy by previous investigations [9]. Therefore, we separated swollen globules manually from the clusters of small globules using the mean plus 3 standard deviation of the area as a reference threshold to form another subtype.

Straight, twisted, and branched tubules were separated by unsupervised clustering into clusters of distinct lengths. However, we assumed that the difference in length only reflects different frequencies of fusion events but not different biological mechanisms of formation. Also, to the best of our knowledge, there is no report in the literature that explicitly distinguishes mitochondrial morphology by length. Thus length was excluded as a criterion to differentiate morphological subtypes. We merged five straight tubule clusters of distinct lengths (Figure 1D) into a single subtype, and similarly merged 3 clusters of twisted tubules (Figure 1E) and 5 clusters of branched tubules (Figure 1F). Twisted tubules have only three instead of five distinct lengths probably because longer twisted tubules have a higher chance to form branched tubules, and that tubules need to reach a certain length before they can become “twisted.” Mitochondria become twisted when they are not fully associated with microtubules, and portions of mitochondria not associated with microtubule motors will collide with water molecules and become twisted. It was observed from our time-lapse images (data not shown) that these twisted mitochondria are constantly moving. We consider only those mitochondria that exhibit these characteristics as “twisted tubules” but not those mildly curved ones.

Finally, we grouped “horseshoe” mitochondria with “donuts” into a single “loops” subtype (Figure 1C), since both of them maintain a much higher degree of stable curvature than twisted tubules. We observed that “horseshoe” mitochondria stably maintain a high degree of curvature, which would require extra force, *e.g.*, inter-mitochondrial end-to-end fusion, to stabilize. In contrast, twisted mitochondria dynamically vibrate their ends due to collision with other molecules, *e.g.*, water, which makes them appear twisted.

### Mitochondrial subtype classification performance

We trained an ensemble of three classifiers on a training set manually labeled with the six morphological subtypes defined in the last section. Performance of the three classifiers were individually assessed both by holdout testing accuracy and visual inspection of results for unlabeled mitochondrial micrographs. Table 1 reports the performance assessment. The figures are averages of 20 runs of holdout testing, where each run used a random partition of training and holdout sets. Table 2 shows the aggregate confusion matrix over all holdout testing runs for all classifiers. Performance for most subtypes are above 80% in accuracy, except for twisted tubules (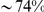), which are often confused with straight tubules by the classifiers. This is expected because it is also difficult for a human to judge whether a short mitochondrial tubule is twisted or straight.

**Table 1:** Holdout test performance of classifiers. Balanced accuracy is the unweighted average of individual accuracies for each subtype. The ensemble classifier is obtained by majority vote of the three other classifiers.

	Accuracy	Balanced Accuracy
Classification tree	89.97%	87.15%
SVM (RBF kernel)	92.00%	88.72%
SVM (linear kernel)	91.93%	88.78%
Ensemble	92.07%	88.97%

**Table 2:** Aggregate confusion matrix of the classifiers in holdout testing.

	Predicted labels					
Correct labels	Small globes	Swollen globes	Straight tubules	Twisted tubules	Branched tubules	Loops
Small globules	97.35%	0.1%	2.37%	0.11%	0%	0.06%
Swollen globules	5.65%	82.83%	6.5%	0.09%	0.07%	4.87%
Straight tubules	4.87%	0.28%	89.6%	4.32%	0.02%	0.92%
Twisted tubules	0.58%	0.09%	20.58%	73.94%	0.15%	4.66%
Branched tubules	0%	0.01%	0.15%	1.86%	96.25%	1.74%
Loops	0.8%	0.55%	6.54%	2.47%	1.18%	88.45%

Next, the entire set of labeled mitochondria samples were used as the training examples for the three classifiers. Table 3 shows the cross validation accuracy results. We also report the optimal parameters used for each classifier. Visual inspection of classification results of unlabeled mitochondria in micrographs shows that all three classifiers performed adequately, with complementary types of errors for each morphology subtype. Hence we used an ensemble classifier, which combines the decisions of the three classifiers by majority vote, as the final classifier for unlabeled cells. The ensemble classifier outperforms individual classifiers in holdout testing.

**Table 3:** Cross-validation performance of classifiers and corresponding optimal parameters. The ensemble classifier is obtained by majority vote of the three other classifiers.

	Cross Validation Accuracy	Optimal Parameters
Classification tree	90.41%	pruning level 28 out of 43
SVM (RBF kernel)	92.21%	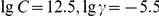
SVM (linear kernel)	92.16%	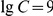
Ensemble	92.28%	same as above

Figures S5–7 in Text S1 show the histograms of basic morphological features of mitochondria for different subtypes. These features are strongly correlated with the subjective criteria used by human inspectors in manual labeling of the training samples.

### Mitochondrial morphological differences of cells from different treatments

Morphological subtyping of mitochondria can help quantify how mitochondrial morphology are affected by drug treatments. Following up on our previous biochemical studies [19], [22], we investigate whether squamocin-induced reduction of mitochondrial biomass and mitochondrial fragmentation are fully restored by z-IETD and z-LEHD. Identifying morphological subtypes may provide new clues for the design of further experiments to determine how these features are correlated with apoptosis.

**Figure 2:**
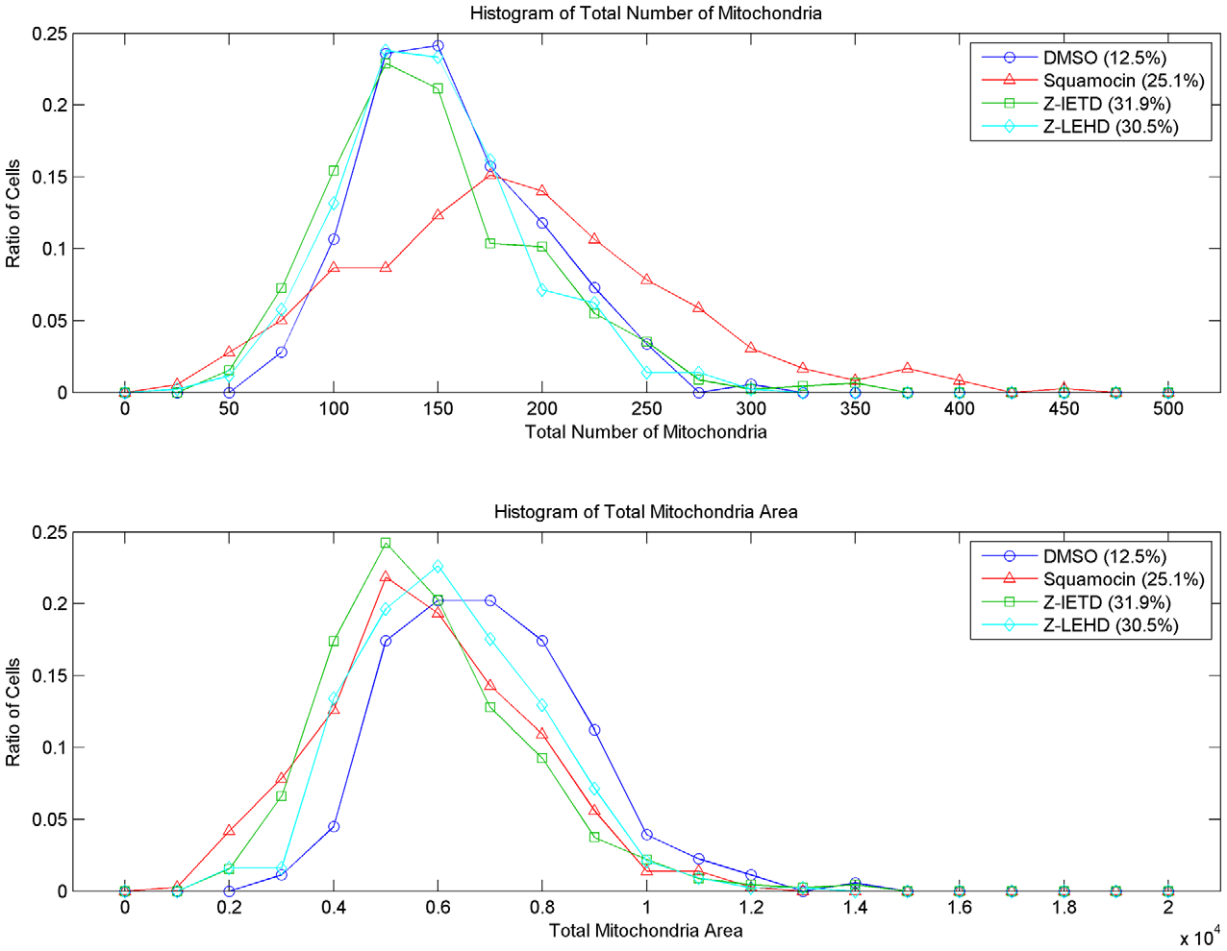
Distribution of the total number and area of mitochondria in individual cells under different treatments. The percentages shown in the legends are the ratio of cells in each treatment population.

Figure 2 shows the effects of different treatments on the total number and area of mitochondria in cells. Squamocin induces large numbers of mitochondria but reduces total mitochondria area compared to control. z-IETD and z-LEHD fully restore the number of mitochondria to the control level but only partially restore the area. Among them, z-LEHD shows slightly better restoration ability than z-IETD. These results suggest that squamocin increases the mitochondria number by inducing mitochondrial fission and possibly also reduces the sizes of individual mitochondria. Squamocin may also reduce biogenesis or enhance degradation of mitochondria, resulting in reduction of total mitochondrial area in cells. In some images of squamocin-treated cells, the intensity of a few mitochondria was lower than the threshold of the segmentation algorithm and thus those mitochondria were omitted and may contribute to reduction of the total area. However, their number is too small to affect the main conclusion here.

**Figure 3:**
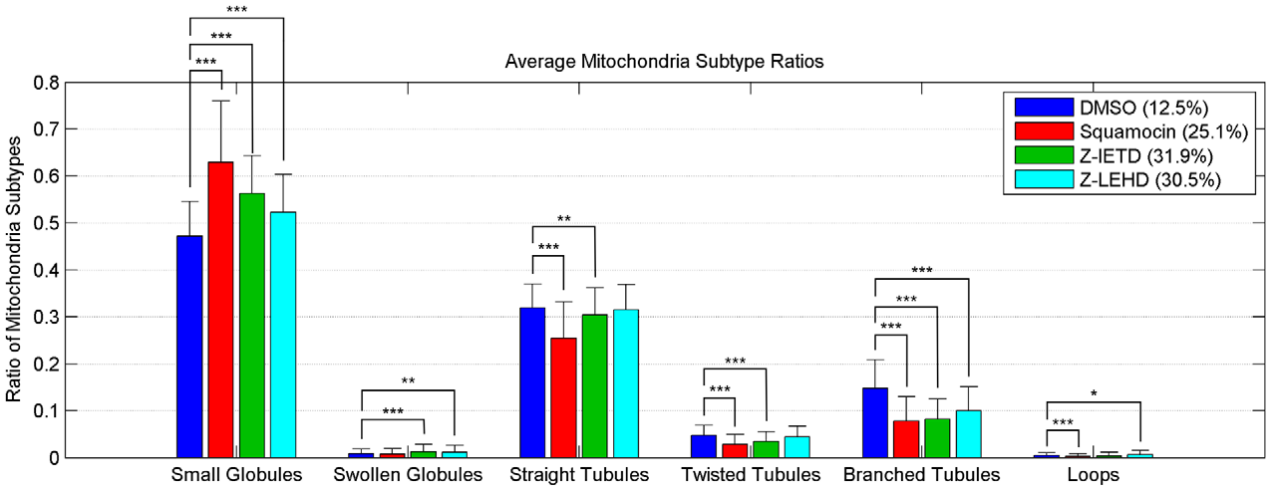
Average ratio of mitochondrial subtypes in individual cells given different treatments. The percentages shown in the legends are the ratio of cells in each treatment population. Error bars represent standard deviation. We performed t-test of differences between control and various treatments, 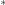 indicates 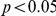, 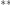 indicates 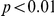, and 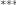 indicates 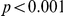.

Figure 3 shows the average ratio of mitochondrial subtypes in cells treated by different drugs. Small globules and straight tubules are predominant in all treatments, followed by branched tubules. The other subtypes are relatively rare. As expected, squamocin induces more small globules and notably decreases the number of branched, twisted tubules and loops. Straight tubules, however, are not affected as much. z-IETD and z-LEHD restore squamocin-reduced straight and twisted tubules completely, but only partially restore branched tubules. Comparing the two Caspase inhibitors, it can be seen that z-LEHD restores more branched tubules than z-IETD, but the difference is not significant. Long error bars (representing standard deviation) in the figure implies that responses of cells to these treatments were quite heterogeneous.

To determine whether the shape of each subtype is affected by different drug treatments, differences in the distributions of mitochondrial morphological features for each subtype was investigated. No significant difference was found for average morphological feature values for cells given different treatments, either over all subtypes or within individual subtypes (data not shown). However, it was observed that tubules were slightly shorter in squamocin-treated cells and that loops were slightly shorter in z-IETD cells. In summary, drug treatments affect the distribution of subtypes in cells but not the shape of the subtypes, although this is expected partly due to the use of the same classifier for all treatment populations.

### Correlations among mitochondrial subtype ratios in different cell populations

**Figure 4:**
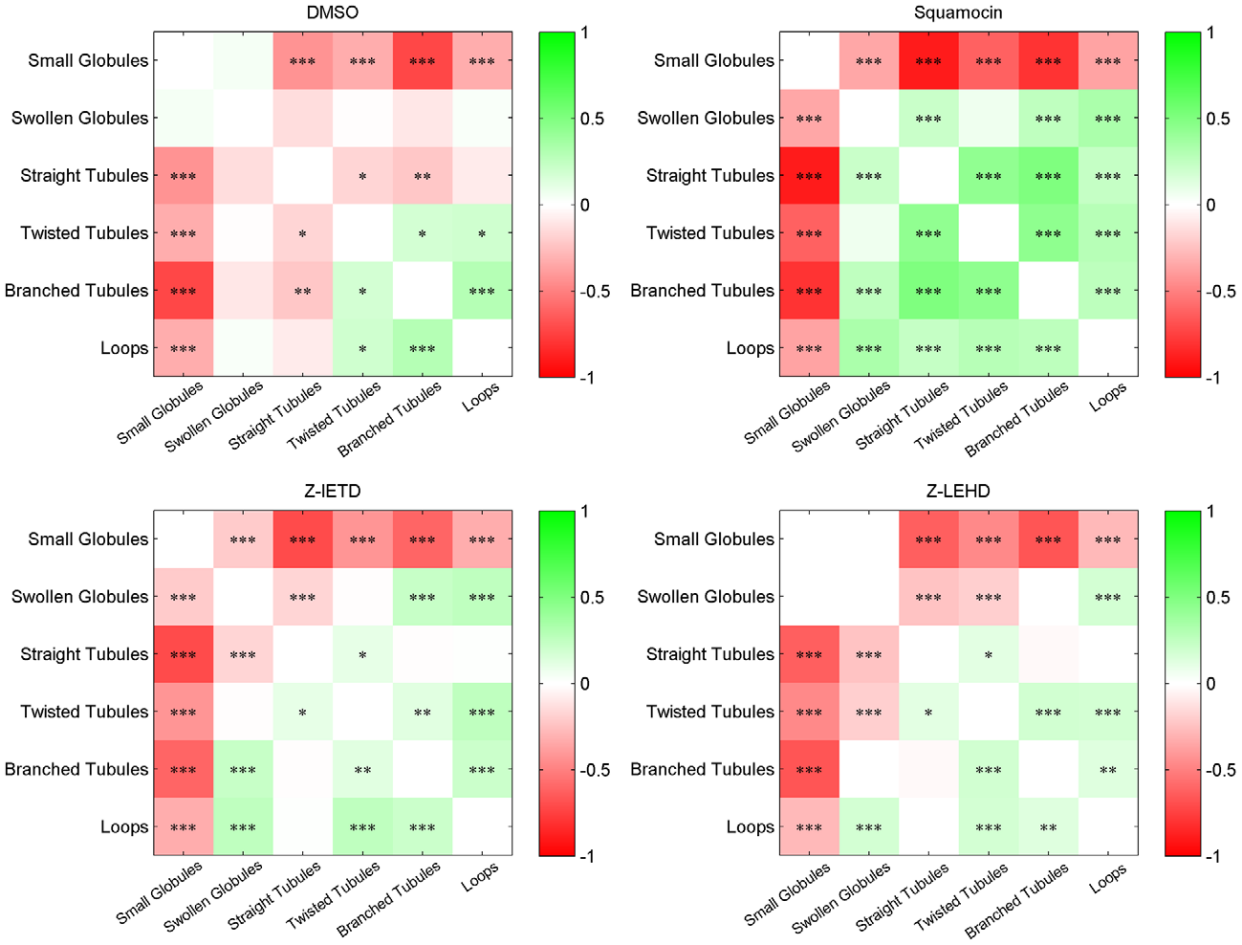
Correlation heat map of the ratios of all pairs of subtypes in cells of different treatments. For each pair, 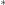 indicates 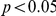, 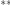 indicates 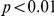, and 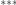 indicates 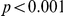. The p-values were computed for the significance of the Pearson's correlation using a Student's t distribution for a transformation of the correlation, as implemented in MATLAB Statistics Toolbox.

The correlations between the ratios of mitochondrial subtypes in each cell may hint at the biological mechanism of their formation. Positively correlated subtypes may be formed by the same mechanism while negative correlation of subtypes implies transition between subtypes. Figure 4 shows the correlation heat map of different subtypes in cells treated by different drugs. The patterns for DMSO (control) and squamocin are quite different whereas those for control and z-LEHD are similar. The correlation pattern of z-LEHD is closer to control than z-IETD, while z-IETD is closer to control than squamocin. This provides further evidence of better restoration capability of z-LEHD. Notable pairwise correlations include:

• Small globules are negatively correlated with other subtypes. This is particularly significant in squamocin-treated cells.
• In squamocin-treated cells, the three tubule subtypes are positively correlated with each other, suggesting that they may share the same formation/fragmentation mechanism triggered by squamocin.
• In squamocin-treated cells, loops are significantly correlated with swollen globules and twisted tubules.
• Branched tubules and loops are positively correlated in all treatments, though the correlation is not as significant in z-LEHD cells.
• Straight and branched tubules are negatively correlated in control cells, in contrast with cells treated by any combination of squamocin and Caspase inhibitors.

### Profiling of cell responses to different treatments

One of the challenges in cell-based analysis is that cells may be at different phases of the cell cycle and have different timing to respond to treatments. The target of this study, mitochondrial morphology, is especially sensitive to bioenergetics, cell cycle, aging, regulatory proteins and various stresses [9]. Therefore, although general trends of squamocin and the restorative effects of Caspase inhibitors can be verified visually, it is challenging to rigorously validate the differences by statistical analysis.

**Figure 5:**
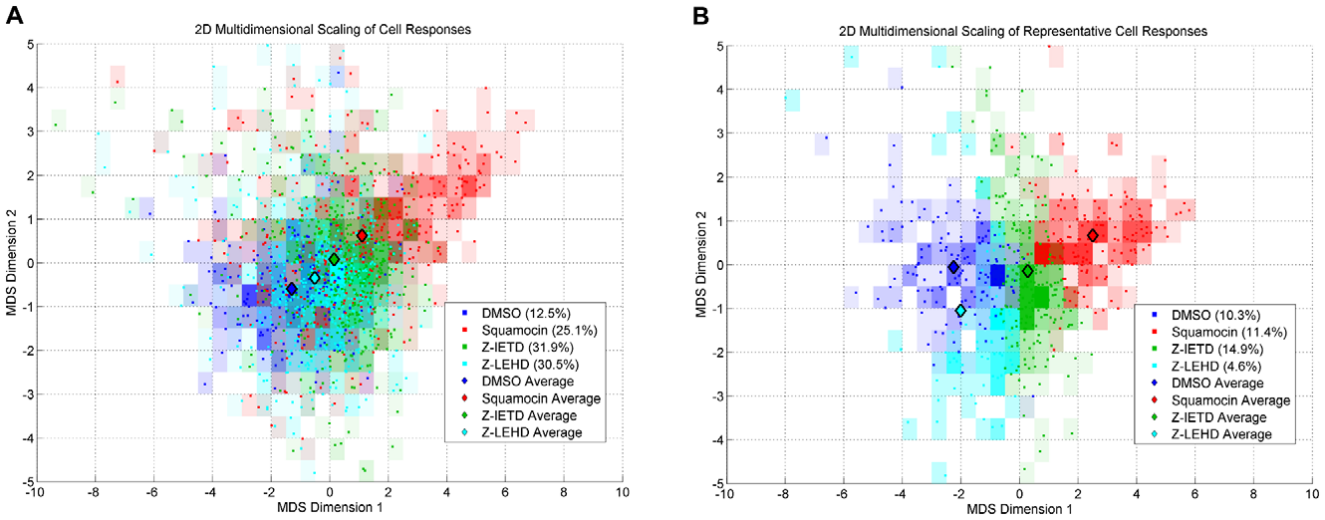
Two dimensional multidimensional scaling (MDS) of cell responses. The percentages shown in the legends are the ratio of cells in each treatment population. (A) MDS of all cells. (B) MDS of representative cells with silhouette coefficients above 0.

For cell response profiling, we used a set of cell features designed to characterize the composition of mitochondrial subtypes in cells. The difference between cells in terms of their mitochondrial morphology can be easily measured as the Euclidean distance in cell feature space. Figure 5A shows the result of multidimensional scaling (MDS) that maps the cells from multidimensional space of cell features to a two dimensional space so that we can visualize their difference. Each point in the figure is a cell that is colored differently, representing the treatment received. The MDS plot shows that cells receiving the same treatment respond heterogeneously and those treated differently overlap. Diamonds in the plot mark the mean responses for each treatment. The distance between the mean responses of Squamocin and DMSO (control) is the largest, implying that responses to these treatments are the most differentiated. Between Squamocin and DMSO lies z-IETD and z-LEHD successively. This result suggests a trend of differing restoration ability of Caspase inhibitors, similar to the results in Figures 2 and 3.

Since both MDS and subtype ratio results show that the responses of cells to the same treatment are heterogeneous and many cells in different treatments have similar responses in terms of mitochondrial morphology, these overlapped responses may be considered as transitional responses or no response. Here, we used the silhouette coefficient analysis [23] to identify cells with representative responses for each treatment. By concentrating on representative cells, a more reliable profiling of these treatments can be established.

**Figure 6:**
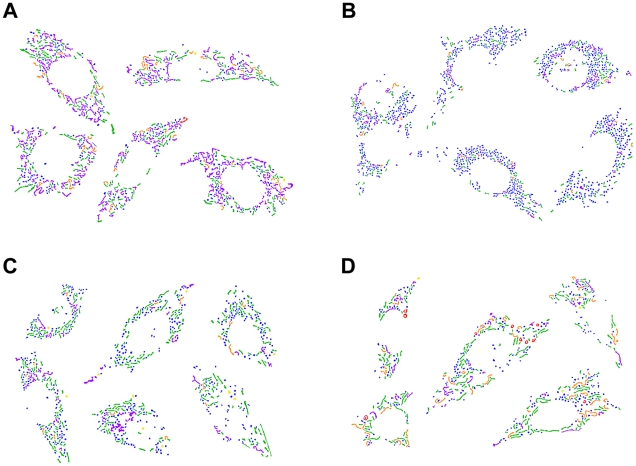
Composition of mitochondrial subtypes in representative cells of each treatment population. The cells were filtered by silhouette coefficient values, individual mitochondria are color-coded as follows: blue - small globules, yellow - swollen globules, green - linear tubules, orange - twisted tubules, purple - branched tubules, red - loops. (A) DMSO. (B) Squamocin. (C) z-IETD. (D) z-LEHD.

We removed cells with silhouette coefficient 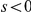 from the analysis, since they are on average closer to cells in another treatment population than to cells in its own treatment population. In the end, the percentages of cells remaining with silhouette coefficient 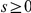 are 82.6%, 45.4%, 46.7% and 15.0% for treatment populations DMSO, Squamocin, z-IETD and z-LEHD, respectively. Figure 5B shows the MDS plot of only these representative cells with the mean responses re-computed. The plot shows that the relative distances between the mean responses stays the same as in Figure 5A, but the relative positions are changed. The subtyping results of representative cells from each population (Figure 6) suggest that in Figure 5B, the x-axis is highly correlated with the degree of fragmentation while the y-axis is highly correlated with the prevalence of twisted tubules and loops. The equivalents of Figures 2, 3, and 4 calculated using only representative cells are shown in Figures S11–13 in Text S1, respectively. These additional results strengthen the support of our main conclusions.

Figure 6 shows subtyping results of select representative cells in different treatment populations. The distributions of the cell features of representative cells (those with 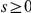) reveal unique characteristics of the effect of the different drugs on the composition of morphological subtypes of mitochondria:

• DMSO (control) cells have more branched mitochondria.
• Squamocin-treated cells have more small globules.
• Cells treated by squamocin and z-IETD have more straight tubules.
• Cells treated by squamocin and z-LEHD have more twisted tubules and loops.

For cells with silhouette coefficient 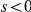, we also keep track of which other treatment population it is closest to, and the percentages of cells in each treatment that are closer to every other treatment populations is shown in Table 4. It can be seen that except for the z-LEHD population, the majority of cells in each treatment is closer to its own treatment population.

**Table 4:** Percentage of cells in each treatment population that is on average closer to cells in another treatment population.

	Percentage of cells closer to another treatment			
Treatments	DMSO	Squamocin	z-IETD	z-LEHD
DMSO	82.58%	2.25%	8.99%	6.18%
Squamocin	30.53%	45.38%	19.05%	5.04%
z-IETD	28.63%	12.34%	46.7%	12.34%
z-LEHD	53.81%	3.93%	27.25%	15.01%

## Discussion

### Biological significance of mitochondrial morphological subtypes

**Figure 7:**
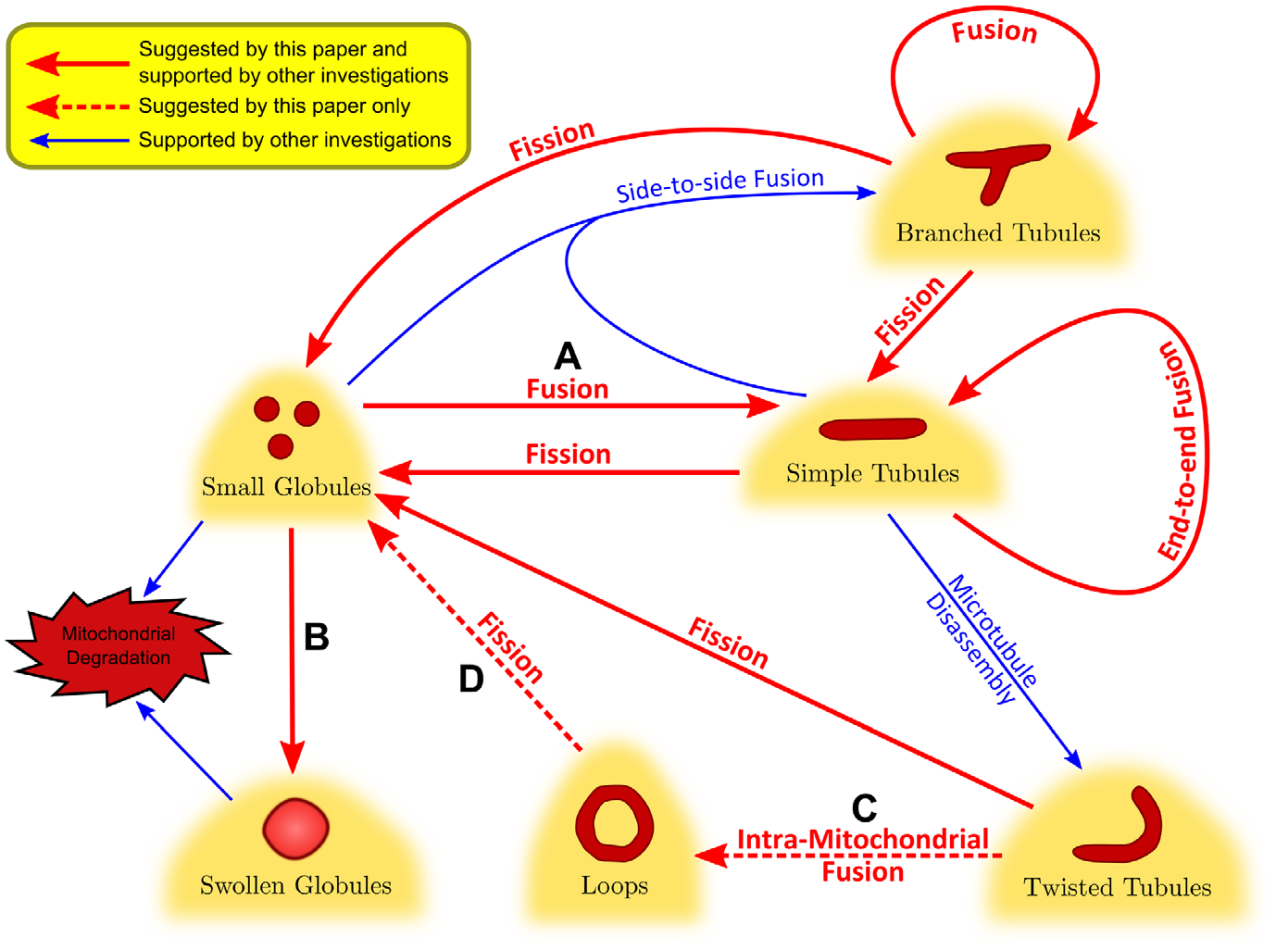
The proposed model for mitochondrial morphological dynamics. (A) Mitochondria form small globules during mitosis and become tubules after cell division [48]. Previous studies discovered that small globules fuse to form tubular mitochondria, this is corroborated by the negative correlation between percentages of small globules and tubules (see Figure 4). (B) Some of the small globules, due to the lack of sufficient genome copies to overcome mutations, are more likely to become dysfunctional and undergo mitophagy to form swollen globules [32], [33]. It is observed in this study that the percentages of small and swollen globules are negatively correlated in squamocin-treated cells (see Figure 4). (C) Percentages of loops and twisted tubules are positively correlated (see Figure 4), suggesting that they might share the same mechanism of formation by microtubule disassembly. (D) In squamocin-treated cells, loops are negatively correlated with small globules. It is possible that squamocin increases Drp1 recruitment to mitochondria, which causes all tubules to be divided into small globules.

The six representative subtypes of mitochondrial morphology: small globules, swollen globules, straight tubules, twisted tubules, branched tubules and loops, are identified based on the unsupervised consensus clustering results and evidence of functional similarity reported in the literature. Based on previous literature and hints from subtype ratio correlations (Figure 4), we propose a model for the formation and transition of the six subtypes (Figure 7).

Previous studies identified three types of mitochondrial morphologies, fragmented, tubular, and network-like mitochondria, and showed that regulatory proteins of mitochondrial dynamics determine which type of mitochondria is predominant [1]. It is also known that bioenergetic states and oxidative stress will affect the distribution of mitochondrial morphology [24]. In our subtyping, fragmented mitochondria are represented by “small globules,” and tubular mitochondria are subdivided into four different subtypes: straight, twisted, branched tubules and loops. Formation of straight or twisted mitochondria are mainly dependent on the assembly of microtubules, but independent of other factors affecting mitochondrial morphology [25]–[27]. Branched tubules result from inter-mitochondrial end-to-end and side-to-side fusion, and has a unique molecular mechanism of formation and distribution specific to physiological conditions [28]–[30]. Donut-shaped mitochondria are induced by moderate mitochondrial membrane potential and low cellular respiration [2], [7], [31]. Network-like mitochondria were not observed in our dataset. Finally, swollen granules are a new unique structure characterized recently. Mitochondria can become swollen due to dysfunction or various conditions (e.g. loss of 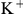/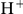 exchange activity), and are subsequently targeted for “mitophagy.” Another formation mechanism involves fragmented mitochondria that are packed into autophagosomes [9]. Recently, Yoshii *et al.* [32] also showed that the outer membrane of mitochondria can be ruptured by mitophagy, resulting in swollen mitochondria.

### Representative mitochondrial morphologies for different treatment conditions

The quantitative analysis in this study demonstrates for the first time that compositions of mitochondrial morphological subtypes may be heterogeneous within a treatment population, with representative morphologies specific to drug treatments. We consider here the biological significance of these representative mitochondrial morphologies for each treatment condition.

The control (DMSO) cells are characterized by tubular mitochondria, especially branched ones (Figure 6A), while small globules are the representative mitochondrial morphology of squamocin-treated cells that are responsive to squamocin (Figure 6B). Table 4 shows that a majority of control cells (83%) are closer to the control population. Hence these cells can be considered to be representative mitochondrial morphology in the control condition. For squamocin-treated cells, 45% are closer to their own population, while 30% are closer to the control population. This indicates that the CHO cells in this research responded heterogeneously when treated by squamocin. Our unpublished data show that longer treatment duration will not change the percentage of cells affected by squamocin and that the effect of squamocin examined in this study is its terminal effect.

Cells treated with z-IETD after squamocin are characterized by straight tubules rescued from squamocin-induced mitochondrial fission, as can be observed from the representative cells in the z-IETD population in Figure 6C. These cells comprise about 47% of the z-IETD population, much larger than the percentage of cells that are closer to other populations. The morphological composition appears to be restored partially to that of the control. In contrast, treatment with z-LEHD after squamocin is more effective for rescuing tubular mitochondria from squamocin-induced fission, as 54% of the z-LEHD population are closer to the control population, while only about 15% are representative. Therefore, the relatively large number of twisted tubules and loops observed in representative cells in the z-LEHD population (shown in Figure 6D) may represent only a transitional morphology during tubule restoration by z-LEHD.

In summary, the mitochondrial morphologies of representative cells in the control, squamocin and z-IETD populations are all unique and specific to their respective treatment conditions. While in the z-LEHD population, a majority of cells are restored by z-LEHD with the mitochondrial morphology similar to control condition. Representative cells in z-LEHD population are the minority and may be special cases of intermediate responses.

### Proposed mechanisms of squamocin-induced mitochondrial morphological changes

Squamocin is a potent inhibitor which blocks mitochondrial functions and causes oxidative stress [15]–. Our results in general show that cells treated with squamocin contain more small globules or fragmented mitochondria and total mitochondrial area is reduced. The squamocin-induced mitochondrial morphological change can be partially rescued by inhibiting Caspases 8 and 9 with z-IETD and z-LEHD treatments, respectively, with z-LEHD being more effective. These results provide the first evidence that Caspases 8 and 9 are directly involved in mitochondrial dynamics. Another interesting finding is that after cells are treated by squamocin, applying inhibitors of Caspases 8 and 9 can rescue most of the tubular mitochondria with the exception of branched ones.

**Figure 8:**
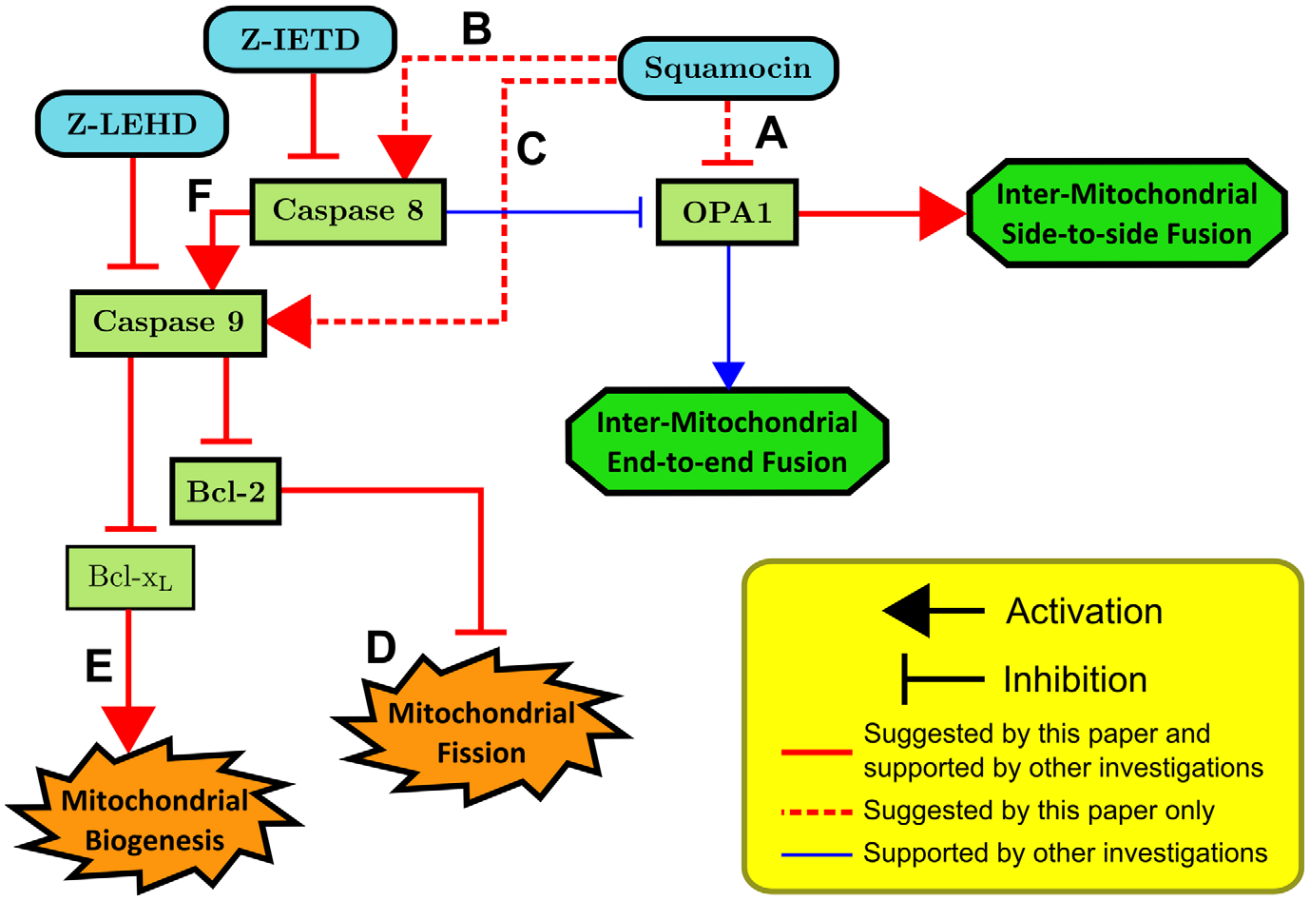
The proposed pathway for the effects of squamocin on mitochondrial morphology. (A) Reduction of mitochondrial membrane potential by squamocin increases cytosolic calcium to activate protease to degrade OPA1, which is a regulatory protein for side-to-side inter-mitochondria fusion [30]. Hence branched tubules are not recovered by Caspase inhibitors (see Figure 3). (B) Our previous data [19] show that squamocin induces nuclear apoptosis, *i.e.* chromatin condensation, can be rescued by Caspase 8 inhibitor. Squamocin may activate Caspase 8 to further inhibit OPA1 or activate Caspase 9 to induce fission and inhibit biogenesis. (C) Squamocin also causes oxidative stress to recruit Bax to mitochondria, which results in cytochrome *c* release. Previous investigations [39], [40] proved that cytochrome *c* release can activate Caspase 9. Therefore, squamocin may also activate Caspase 9 via oxidative stress. (D) In this study, it is observed that percentages of small globules and tubules are negatively correlated (see Figure 4), providing further evidence that tubular mitochondria fragments into small globules. (E) Squamocin reduces expression of 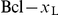, which is an essential protein for mitochondria biogenesis [10], via Caspases 8 and 9, causing reduction of total mitochondria area of individual cells (see Figure 2). (F) Caspase 8 cleaves Bid, and cleaved Bid recruits Bax to mitochondria. Bax proteins form pores on mitochondrial outer membrane, and release cytochrome *c* and subsequently activate Caspase 9 [4].

According to our analysis and previous investigations, we proposed a hypothetical model of squamocin-induced mitochondrial morphological changes, as shown in Figure 8. We discuss these results in more detail in the following sections.

#### Molecular mechanism of mitochondrial area reduction induced by squamocin

Two major pathways may possibly lead to the reduction of total mitochondrial area induced by squamocin. The first possibility is that squamocin reduces total mitochondrial area via mitophagy (*i.e.* autophagy of mitochondria). Some of the small globules, due to the lack of sufficient genome copies to overcome mutations, are more likely to become dysfunctional and undergo mitophagy to form swollen globules [32]–[35]. Dagda *et al.* [36] showed that formation of small globules is essential for mitophagy, which was observed in our result of correlation between ratios of small and swollen globules after squamocin treatment (Figure 4). Squamocin may suppress complex I or activate Caspases to trigger mitochondria to fission into small and swollen globules, which tend to degrade via mitophagy, resulting in reduction of the total area of mitochondria.

The second possibility is that mitochondrial biogenesis regulated by 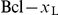 is inhibited by squamocin through the activation of Caspases 8 and 9 to decrease the total area of mitochondria. Our biochemical analysis showed that squamocin suppresses expression of 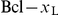 (unpublished results). Mitochondrial biogenesis is known to be regulated by 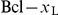 [10], which can be degraded or inhibited by Caspases [37], [38].

#### Molecular mechanism of mitochondrial fission induced by squamocin

Current investigations on apoptosis and mitochondria suggest that oxidative stress, an extrinsic apoptotic signal, results in recruitment of Bax and Drp1 to mitochondria to cause mitochondrial fission, while mitochondrial fission increases release of cytochrome *c* to activate Caspase 9 [39], [40]. However, our results show that both Caspase 8 and 9 inhibitors rescue mitochondria from squamocin-induced fission, suggesting that activation of Caspases 8 and 9 may cause mitochondrial fission.

Combining our results with those from Yuan *et al.* [18], we hypothesize that squamocin activates Caspases 8 and 9 to further activate Caspase 3, which in turn degrades Bcl2 [41]. Our biochemical data showed that squamocin reduces Bcl2 expression to recruit Bax to mitochondria [19]. Because Bax can bind Drp1, a positive regulatory protein for mitochondrial fission, recruitment of Bax to mitochondria at low Bcl2 expression level has been proved to induce mitochondrial fission [40]. Therefore, it is likely that squamocin first activates Caspase 8, which inhibits Bcl2 via Caspase 9 to further induce mitochondrial fission [41]. Loucks *et al.* [42] also showed that Caspase 8 can directly inhibit OPA1, a positive regulatory protein for mitochondrial fusion.

Given the discussion above, we propose that Caspase 8 can be activated by squamocin to directly inhibit OPA1 or inhibit Bcl2 via Caspase 9 (see Figure 8), resulting in more short straight tubules and small globules due to their effects on mitochondrial fusion and fission. Further biochemical experiments are required to verify whether Caspase inhibitors can fully recover OPA1 concentration or suppress recruitment of Bax and Drp1 to mitochondria.

#### Effectiveness of Caspase 9 inhibitor at rescuing squamocin-induced mitochondrial changes

Our previous results [19] and results reported in [40] showed that, as an inhibitor of complex I, squamocin may activate Caspase 9 via the intrinsic apoptosis pathway without activating Caspase 8. As illustrated in Figure 8, at the downstream of Caspase 8 in the pathway, Caspase 9 can be activated via both intrinsic and extrinsic pathways. As a result, Caspase 9 can trigger mitochondrial fission when Caspase 8 is inhibited. On the other hand, when Caspase 9 is inhibited, the upstream Caspase 8′s role in promoting fission would be blocked. Therefore, Caspase 9 inhibitor is more effective than that of Caspase 8 for rescuing mitochondria from fragmentation.

#### Ineffectiveness of Caspase inhibitors at rescuing squamocin-induced fission of branched tubules

Figure 2 shows that even Caspases 9 inhibitor cannot restore the ratio of branched tubules to as high as that in control cells. Our proposed pathway model (Figure 8) explains why this is the case. First, besides activation of Caspase 8, reduction of mitochondrial membrane potential can also reduce OPA1 concentration by activating protease to degrade OPA1 [2]. OPA1 concentration is crucial for mitochondrial branching [30]. Low OPA1 concentration facilitates formation of linear mitochondrial tubules (via end-to-end fusion), while high OPA1 concentration allows formation of branched tubules (side-to-side fusion). Because squamocin can still inhibit mitochondrial membrane potential when Caspases 8 and 9 are blocked, formation of branched tubules will not be rescued by the treatment of Caspase inhibitors [19]. Besides, as we discussed earlier, when only Caspase 9 is inhibited, Caspase 8 can still directly inhibit OPA1 [42], which may also contribute to our observations. Second, as reported by Legesse-Miller *et al.* [43], fission of mitochondria occurs preferentially at branch points of mitochondria. Without activating Caspases 8 and 9, squamocin can still induce mild fission to break down branched tubules by reducing mitochondrial membrane potential. Our correlation analysis shows that in all treatment groups, ratios of tubule subtypes are all negatively correlated with small globules (Figure 4). Among these negative correlations, the ones for branched tubules are usually the highest, suggesting that branched tubules tend to undergo fission into small globules.

#### Cell viability and mitochondrial morphology

Previously, we observed that cells treated with squamocin exhibit decreased viability compared to control, and pre-application of Caspase inhibitors did not increase viability. We speculated that Caspase inhibitors may not have fully restored mitochondrial structures even though fragmentation level of mitochondria was reduced [19]. Our numerical results show that mitochondria in cells treated by Caspase inhibitors and squamocin revealed a wide variety of morphological compositions. In particular, the results suggest that inhibition of Caspases does not affect squamocin-induced reduction of mitochondrial membrane potential, resulting in ineffectiveness of Caspase inhibitors at rescuing squamocin-induced fission of branched tubules. On the other hand, Table 4 shows that z-LEHD restored about 20% more cells back to the control condition than squamocin. How these morphological conditions correlate with cell viability is an interesting question. The answer will further reveal the relation between squamocin-induced apoptosis and mitochondrial morphological changes and will be the aim of our future study.

### Conclusions and future work

In this study, we developed MicroP for automatic classification and quantification of mitochondrial morphology in cell micrographs, which helped us confirm the number and range of representative morphological subtypes, the effects of treatments on the ratio of subtypes in cells, and make sense of the subpopulations within heterogeneous cell responses to different drug treatments.

The main contributions of our automated system in this study are summarized as follows: First, our computational method allows objective subtyping and automatic quantification of mitochondrial morphology in cell micrographs, which enables profiling of cell responses to drug treatments. Second, using multidimensional scaling and silhouette coefficients to characterize cell response profiles, we discovered that Caspases suppress elongation fusion of mitochondria but not branching. Our quantification analysis also differentiates the effects of Caspases 8 and 9 inhibitors on squamocin-treated cells. For example, Caspase 9 inhibitor (z-LEHD) rescues longer mitochondria and results in larger numbers of twisted and looped mitochondria than Caspase 8 inhibitor (z-IETD). Given the heterogeneity of cell responses to drug treatments, it would be challenging to reach these findings if not for the subtyping and quantitative analysis. Finally, our correlation analysis of subtype ratios within individual cells reveals unexpected trends which provide directions for further investigations. For example, the stronger negative correlations of branched tubule mitochondria with small globules than those of other tubule subtypes suggests a higher fission rate of branched mitochondria than other subtypes of tubular mitochondria.

Our future work is to simultaneously measure cell viability and morphological subtypes of mitochondria to reveal their correlation and to validate the proposed pathway model with further biochemical and cell biological experiments. We will utilize 3D time-lapsed imaging to provide solid evidence to study the transition of mitochondrial subtypes during different treatments, and to verify our interpretations of the correlations of mitochondrial morphological subtypes. Our long-term goal is to investigate whether morphological features of mitochondria are specific to neurodegenerative diseases or aging and evaluate the use of mitochondrial morphological features as “high content” biomarkers.

## Materials and Methods

### Cell culture and drug treatments

CHO-K1 cells were obtained from the Food Industry Research and Development Institute (Hsinchu, Taiwan). CHO-K1 cells were cultured in McCoy' 5A containing 10% fetal bovine serum and incubated in an incubator containing 5% 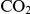 at 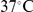. For cell imaging, CHO-K1 cells expressing DsRed-mito (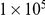 cells) were seeded on a 24mm round coverslip (thickness: 0.17mm). After 24h culture, cells were pre-treated with 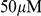 z-IETD (Caspase 8 inhibitor; Sigma, U.S.A.), z-LEHD (Caspase 9 inhibitor; Sigma, U.S.A.), or a control medium without any drugs for 2h. Cells pre-treated with control medium were further incubated with either 0.05% DMSO or 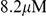 squamocin for 24h. Cells pre-treated with Caspase inhibitors were further treated with 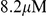 squamocin for 24h. The following labels are used to refer to the different treatments in subsequent sections: DMSO (DMSO + control), Squamocin (squamocin + control), z-IETD (Squamocin + z-IETD) and z-LEHD (squamocin + z-LEHD).

### Cell imaging

The coverslip attached with cells was put on a chamber and examined with a fluorescence microscope (IX-71, Olympus) with a 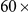 objective (PlanApo, NA1.45, Olympus). Monochromator equipped with Xe-lamp (polychrome II, Till-photonics, Gräfelfing, Germany) was driven by TILLVision 4.0 (Till-Photonic, Gräfelfing, Germany) to excite DsRed-mito (550 nm). Fluorescence was filtered by a filter cube (Mitotracker orange: 565DCLP (BS), D605/55m (Em); Chroma, Rockingham, Vermont, USA), and 2D fluorescent cell images were acquired by a CCD camera (IMAGO, Till-Photonics Germany; exposure: 500ms; resolution: 12-bit, 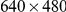 pixels, pixel size: 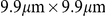). The images are scaled down to 8-bit for image analysis, and pixel width is about 165nm.

We have compared 2D (epi-fluorescence microscopy) and 3D (confocal fluorescence microscopy) CHO micrographs, and found that CHO cells are flat and most of the mitochondria in 2D micrographs are in focus and clear enough for high content image analysis (see Section S2.1 in Text S1). Therefore, 2D micrographs are the major data source used in this study.

### Cell segmentation

Segmentation of individual cells within each micrograph field was done semi-automatically. First the centroid of each cell nucleus was specified manually, which indicated the position of each individual cell object. Then the Delaunay triangulation was calculated using all cell centroids and their dual Voronoi diagram [44] was used as the final cell segmentation. The resulting single-cell images were manually validated and grouped by drug treatments. In the resulting set, the DMSO group contains 178 cell images, the Squamocin group 357 cell images, the z-IETD group 454 cell images, and z-LEHD group 433 cell images, for a total of 1422 single cell images.

### Mitochondria extraction

Mitochondria extraction involved segmenting each single-cell image into mitochondria and background. Cell micrographs of mitochondria exhibit varying background brightness and contrast levels, hence proper segmentation needs to take into account the statistical properties associated with each locality in the image. Here, we used our segmentation algorithm described in [20]. In this algorithm, adaptive local normalization is used to preprocess the images (with parameter value 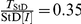) and then Otsu's thresholding [21] was applied to the normalized image to obtain the final segmentation. Adaptive local normalization applies dynamic window sizes determined by the intensity structure of each pixel region to effectively deal with local contrast and background variation, and at the same time enhance detailed subcellular structures. This segmentation algorithm was quantitatively validated using manually generated gold standard segmentations from the same dataset used in this work with approximately equal amount of images from each treatment [20].

All the CHO cells from the four treatment conditions were quite flat and easily focused on a 2D focal plane. Seriously out-of-focus and blurred mitochondria generally only constituted a small fraction of all mitochondria in individual cells. Cells treated by squamocin were a little bit rounder, but their mitochondria were still focused well on a single plane (as shown in Figures S14-16 in Text S1). Visual examination showed that cells from four treatment conditions were roughly equally focused on average, and there were no seriously out-of-focus cells in this dataset. To further test the robustness of our segmentation method, we analyzed representative subsets of cells from each treatment condition with perturbed threshold values (data not shown). For most cell features, such as total mitochondrial area and relative ratios of morphological subtypes, the results are consistent across different threshold values.

After segmentation, binary object images representing mitochondria were extracted by standard object labeling with 4-neighbor connectivity. Postprocessing was performed to remove objects with low intensity (both in the original or normalized images) and small area, as well as any objects touching the image boundary. Figure S17a-c in Text S1 illustrate the results of these processing steps on an example micrograph.

A total of 225,556 objects/mitochondria were obtained from segmenting the single-cell images, of which 27,752 are in the DMSO group, 66,438 in the Squamocin group, 67,288 in the z-IETD group, and 64,078 in z-LEHD group.

### Feature extraction from mitochondrion objects

From each segmented binary mitochondrion object, a set of image features was extracted to represent its morphology. These image features can be divided into three categories: morphological features based on the object binary mask, skeleton features based on one-pixel wide homotopic skeleton, and binary texture features based on the object bounding convex hull. Table S1 in Text S1 contains a summary of these features and their notations, for more details please refer to Section S2.3 in Text S1. These mitochondrial features are used for both consensus clustering and classification, described in subsequent sections.

### Clustering of mitochondria

To identify meaningful morphological subtypes of mitochondria, the Gaussian mixture model (GMM) clustering was applied to cluster mitochondrial objects with the optimal number of clusters 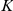 determined by Bayesian information criterion (BIC). We used the GMM implementation in the MATLAB Statistics Toolbox with default values for all parameters. We performed multiple clustering runs on sampling-with-replacement random subsets of objects to ensure the robustness of the clustering. Processing small subsets also helped us save computational cost due to both large data size and slow convergence. For each run, a random subset of 10% of all objects was chosen, then GMM clustering was performed with 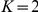 to 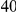, and the 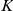 value with a minimum BIC was chosen as the optimal number of clusters for the subset. Feature transformation (see Section S2.4 in Text S1) was performed for each sampled subset before clustering. Clustering results from multiple runs were compared and combined by matching up clusters with similar morphology so that well-represented morphologies of sufficient diversity would be discovered.

Matching clusters from different clusterings are done by calculating the average distance between objects in the clusters. The distance between two clusters 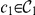 and 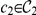 from two different clusterings 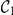 and 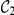 are defined as the average distance over all pairs of objects, 
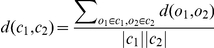
, where 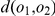 are the Euclidean distance of the mitochondrial feature vectors of 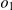 and 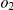. Two clusters 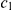 and 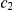 are considered a match if both 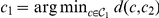, and 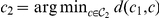. Eventually, we obtained a final set of 19 clusters. These matched clusters were visually verified by human experts as reasonable and labeled with biological significance.

### Classification of mitochondria

Using the set of morphology clusters of mitochondria identified by the above process, we defined a set of six distinct and meaningful mitochondria subtypes. We then developed an automated classification system to classify a mitochondria object into one of the six subtypes.

The training data set for the classification system was generated by manually labeling the morphological subtypes. Example images in the subtype clusters found by consensus clustering were used as visual references for labeling. A total of 10662 mitochondrial objects were labeled as the gold standard for training, validation and evaluation of the classification system. The gold standard contains 5183 small globule examples, 190 swollen globules, 2923 straight tubules, 961 twisted tubules, 693 branched tubules, and 712 loops. The proportion was made to reflect that of the observed population of mitochondria, and adjusted to have sufficient training samples for each subtype.

We used an ensemble of three classifiers trained by three supervised learning methods, respectively. These methods include decision trees, support vector machines (SVM) with a radial basis function kernel, and SVM with a linear kernel. The final decision of the ensemble classifier is determined by majority vote. We applied a coarse-to-fine grid search to optimize hyperparameters for the SVM with stratified 10-fold cross validation accuracy as the criterion. We extended binary SVM to multi-class classification using the “one-against-one” approach [45], in which 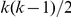 classifiers are constructed to classify an input into 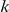 classes. Each classifier is trained to distinguish two of the 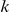 classes, as implemented in LIBSVM [46].

For the decision tree method [47], the MATLAB Statistics Toolbox implementation was used. A full tree was first constructed from the training data and optimal pruning level determined by stratified 10-fold cross validation (via a complexity measure as implemented in MATLAB). Misclassification cost was defined as one for each error and zero for a correct classification.

The feature set used was the same as the one for the consensus clustering. No feature selection was performed since there are only about 20 features and both SVM and decision trees are relatively robust against redundant features. Also most feature subsets obtained using forward selection failed to improve classifier performance (data not shown). Performance of the classifiers were assessed by holdout testing containing 50% of the gold standard selected by stratified sampling. The remaining 50% were then used as training and validation sets for both optimization and training of the classifiers. This process was repeated for 20 times to obtain an aggregate final accuracy rate for each classifier and the ensemble. Figure S17(d) in Text S1 shows a typical example of the mitochondria classification.

### Cell feature profiling

We used the trained ensemble classifier to automatically classify mitochondria in each cell into one of the six subtypes, and calculated a set of cell-level features based on the distribution of mitochondrial subtypes in each cell. These features include total number and area of mitochondria, and the ratio of each subtype in a cell (see Table S2 in Text S1 for the complete list of cell features). These cell features represent the mitochondrial morphology characteristics within a cell.

To characterize the distribution of cells in the feature space, we used multidimensional scaling (MDS) to map the cells onto a 2D space to visualize their distribution. We used the MDS implementation in the MATLAB Statistics Toolbox and performed non-metric MDS using the Euclidean distance of transformed feature values as cell distance, and the stress loss function normalized by the sum of squares of the inter-point distances as the goodness-of-fit criterion.

To find representative cell responses, we first divided the cells into four clusters according to treatment received, and the silhouette coefficient of each cell was computed to estimate the representativeness of its responses among all cells receiving the same treatment. The silhouette coefficient is defined for an object 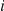 in cluster 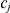 by



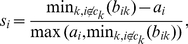
(1)where 
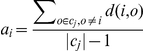
 is the average distance from object 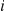 to every other objects in the same cluster, and 
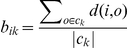
 is the average distance from object 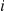 to every object in cluster 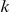. So when 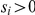,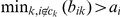, the average distance of object 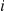 is the shortest to objects in its own cluster, hence it can be viewed as a “representative” object in its cluster. Conversely, when 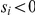, average distance to another cluster is the smallest for object 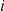 than to objects in its own cluster, hence it cannot represent the cluster that it belongs to. Here we chose 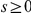 as the threshold for a cell to be representative in its treatment population.

## Supporting Information

Text S1 Supplementary Results and Methods.
(PDF)
