# Supplementary material for: Automatic Morphological Subtyping Reveals New Roles of Caspases in Mitochondrial Dynamics
Source: PLoS Comput Biol. 2011 Oct 6;7(10):e1002212. doi: 10.1371/journal.pcbi.1002212 (PMC3188504; doi:10.1371/journal.pcbi.1002212)
Supplement: Text S1 — Supplementary Results and Methods. (PDF) [file pcbi.1002212.s001.pdf]

Automatic Morphological Subtyping Reveals New Roles of Caspases in Mitochondrial Dynamics - Supplementary Text S1

Jyh-Ying Peng, Chung-Chih Lin, Yen-Jen Chen, Lung-Sen Kao, Young-Chau Liu, Chung-Chien Chou, Yi-Hung Huang, Fang-Rong Chang, Yang-Chang Wu, Yuh-Show Tsai and Chun-Nan Hsu

S1 Supplementary Results

S1.1 Mitochondria segmentation and classification results on cells from different treatments

Figures S1-S4 show segmentation and classification results of select cells from each treatment population.

S1.2 Comparison of morphological features of different mitochondrial subtypes

Figures S5-S7 show the feature histograms of mitochondria for different subtypes. The percentages shown in the legends of these figures are the ratio of each subtype in the data set. A majority of small globules has area about 25 pixel^2, BWCM energy mean and homogeneity mean equal to 1, and BWCM contrast mean equal to 0. Swollen globules have an area larger than 50 pixel^2 and axial ratio ≤ 2. Loops are characterized by Euler number ≤ 0, while for all other subtypes, the Euler number $= 1$. Branched tubules are characterized by having one or more branch points. The majority of straight tubules has axial ratio ≥ 2 and minor axis length $= 5$ pixels. These features are strongly corre-

lated with the subjective criteria used in expert manual labeling, and are informative for automatic subtype classification of the corresponding subtypes.

S1.3 Area distributions of mitochondria from different morphological subtypes

Figure S8 shows mitochondria area distributions for different mitochondrial subtypes.

S1.4 Histograms of mitochondrial subtype ratios in cells from different drug treatments

Figure S9 shows histograms of mitochondrial subtype ratios in cells from different drug treatments.

S1.5 Cell Treatment Cluster Silhouettes

Silhouette plot of cells using treatment population as clusters is shown in Figure S10. The histograms of total number and area of mitochondria, average subtype ratio bar plot, and subtype ratio correlation heat maps calculated using only representative cells (those with silhouette coefficient above 0) are shown in Figures S11, S12, and S13, respectively.

S2 Supplementary Methods

S2.1 Comparison of 2D epi-fluorescence microscopy with 3D confocal fluorescence microscopy

Figures S14-S16 show comparison between 2D and 3D imaging of control (DMSO) and squamocin-treated cells. In each figure, the left panel shows a slice of confocal micrograph at the same z-axial position (indicated by a thin blue line) as that in the 2D micrographs (right panel) with orthogonal views. In the left panel, the horizontal green line indicates

the x-z section projected in green box and the vertical red line indicates the y-z section projected in red box. In all micrographs, the red channel is the mitochondria marker, and blue channel is the nuclei marker.

Almost all CHO cells observed in our dataset are very flat (2-3μm), and even squamocin-treated cells are still flat when their mitochondria are fragmented or form donuts (Figure S15-S16). The figures show that the focal planes covering most of mitochondria in 2D micrographs are close to the coverslip (the blue line in 3D confocal micrographs), and it can be seen that most of mitochondria are at the blue lines, i.e. the focal plane used in 2D imaging. Thus 2D imaging is enough to provide almost correct and complete information for our current research.

S2.2 Image analysis results on an example cell micrograph

Figure S17 illustrates our image processing procedure on an example micrograph.

S2.3 Mitochondrial Feature Definitions

Table S1 shows the numerical features used to represent individual morphologies for mitochondria. There are three groups of features: standard morphological features, skeleton features, and binary texture features.

The morphological features include area a, perimeter p, compactness $p^2/(4\pi a)$, major/minor axis length and ratio, solidity w.r.t. the bounding convex hull area, a_{cv}, and Euler number, which is defined as the total number of objects in an image area minus the number of holes in those objects. The skeleton features include skeleton length l_s (the number of skeleton pixels), skeleton ratio l_s^2/a, principle axis length l_c (the shortest path length between the two farthest endpoints of the skeleton), principle axis ratio l_c^2/a, number of skeleton branch points, and the proportion of skeleton pixels that form loops. When counting the number of branch points, trivial one-pixel branches were excluded and adjacent branch point pairs were counted as one. Previously, skeleton features were

shown to be effective for classifying subcellular patterns [1].

The binary texture features are Haralick texture features [2] calculated using the object mask, based on gray level co-occurrence matrices with only two intensity levels, which is referred to as black-and-white co-occurrence matrices (BWCM). Only the pixels inside the bounding convex hull of the object are used, which adds some degree of rotational invariance to the features calculated. Specifically, the mean and range of energy, homogeneity and contrast over four directions with offset 1 are calculated as the features, using the object mask within the bounding convex hull of the object.

All feature calculation routines are implemented in `MATLAB` using the `Image Processing Toolbox`.

S2.4 Feature Transformation for Exploratory Clustering

The feature values were transformed by independent normalization or re-scaling prior to clustering. Normalization was performed for each feature independently to produce transformed features with zero mean and unit variance, with feature values higher/lower than the mean plus/minus 3 standard deviations set to the mean plus/minus 3 standard deviations. Re-scaling was performed by first setting all feature values higher than the 99th percentile to the 99th percentile and those lower than the 1st percentile to the 1st percentile, and then scaling the feature values to the range $[0, 1]$ linearly.

S2.5 Preliminary Clustering Analysis of Mitochondria

A preliminary analysis using k-means clustering is performed on all objects with the number of clusters $K = 4, 7$ and 10 (data not shown), using `MATLAB Statistical Toolbox`. Clusters are visually compared and distinctive morphologies are identified. From these preliminary results (data not shown) it can be seen that object features such as elongation and branching complexity are "quantized" by the clustering algorithm, but each cluster contains heterogeneous morphologies, indicating that the number of clusters K should

be set higher. We instead applied GMM with BIC for the final analysis so that we can objectively determine K and obtain more representative clusters.

S2.6 Cellular Feature Definitions

Table S2 shows numerical features used to characterize mitochondrial morphology for individual cells.

References

[1] Robert F. Murphy, Meel Velliste, and Gregory Porreca. Robust numerical features for description and classification of subcellular location patterns in fluorescence microscope images. *The Journal of VLSI Signal Processing*, 35(3):311–321, Nov 2003.

[2] Robert M. Haralick, K. Shanmugam, and Its'Hak Dinstein. Textural features for image classification. *IEEE Transactions on Systems, Man and Cybernetics*, 3(6):610–621, Nov 1973.

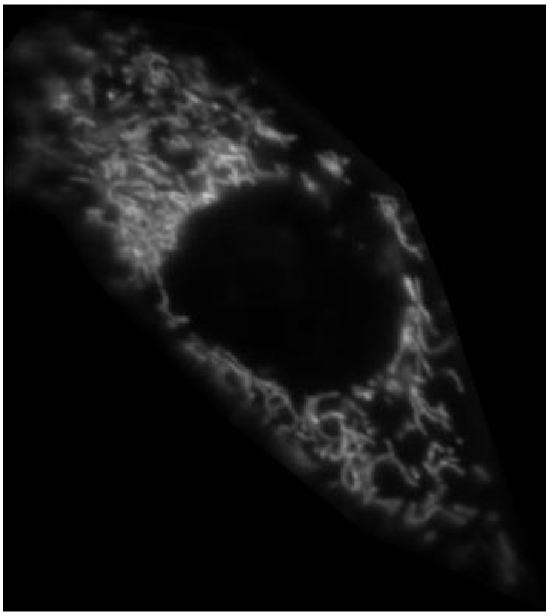

(a) Original image.

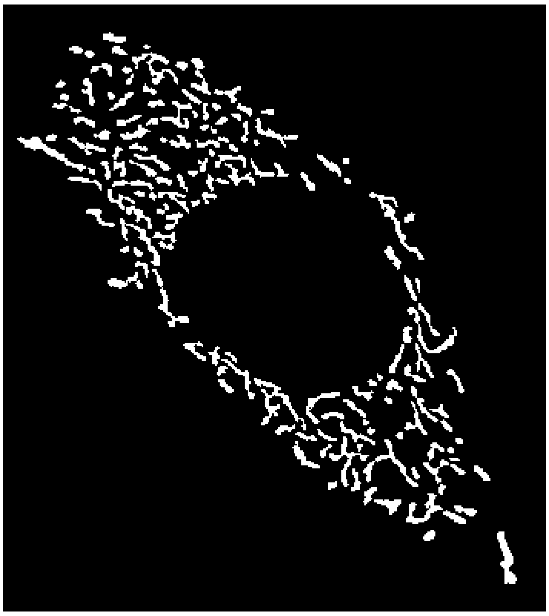

(b) Segmented image.

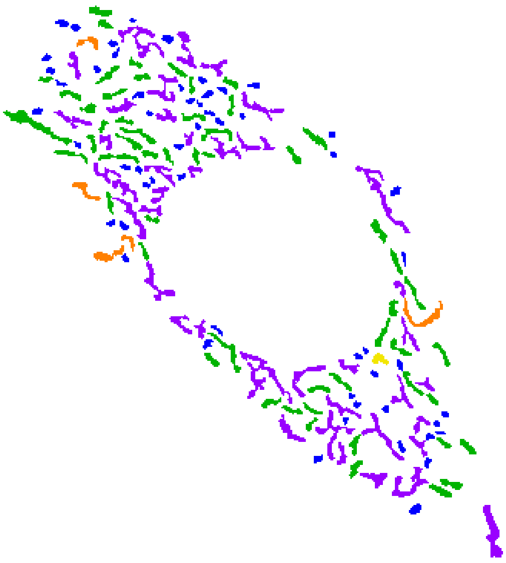

(c) Object classification image.

Figure S1: Image processing results of a cell from DMSO treatment. See Figure S17 for mitochondria subtype color codes.

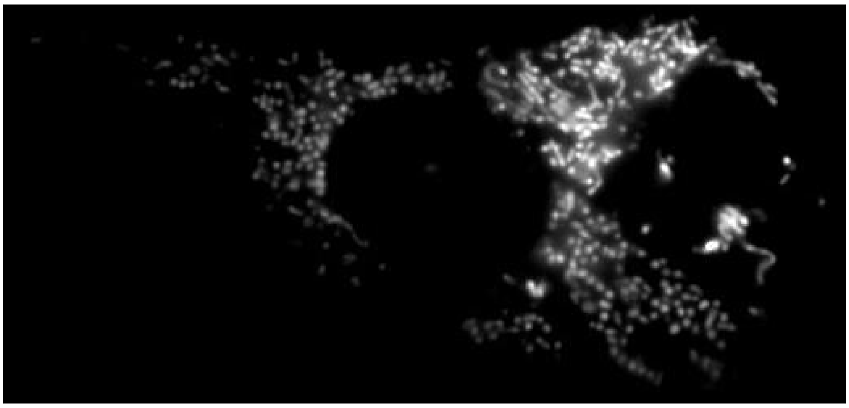

(a) Original image.

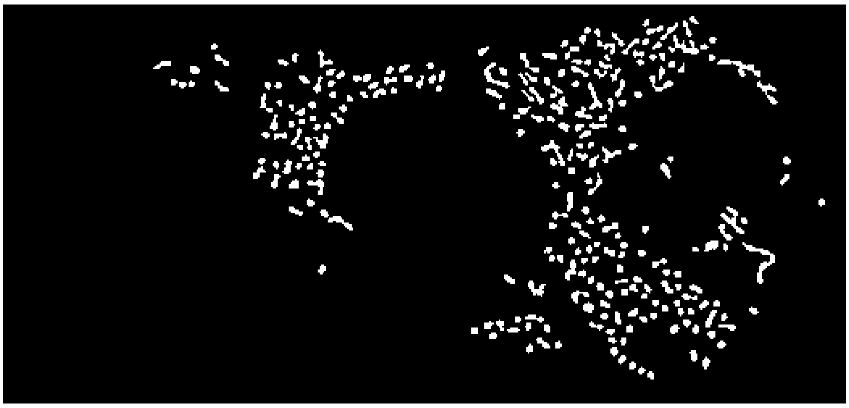

(b) Segmented image.

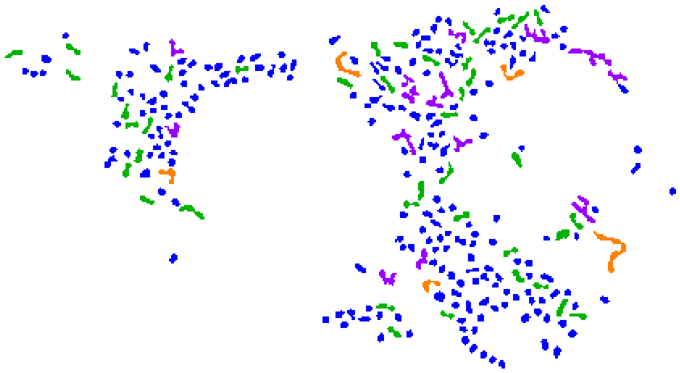

(c) Object classification image.

Figure S2: Image processing results of a cell from squamocin treatment. See Figure S17 for mitochondria subtype color codes.

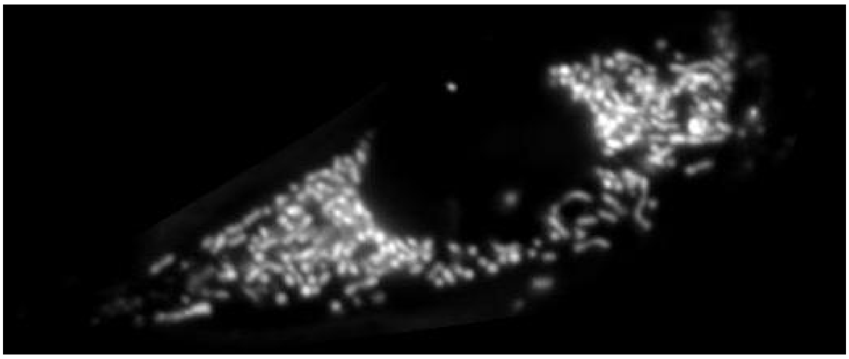

(a) Original image.

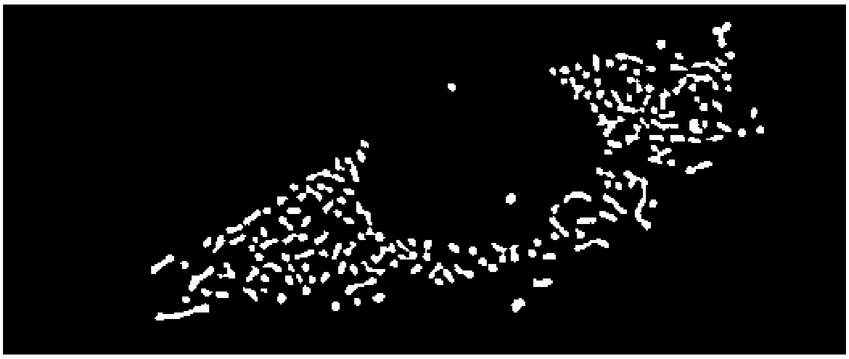

(b) Segmented image.

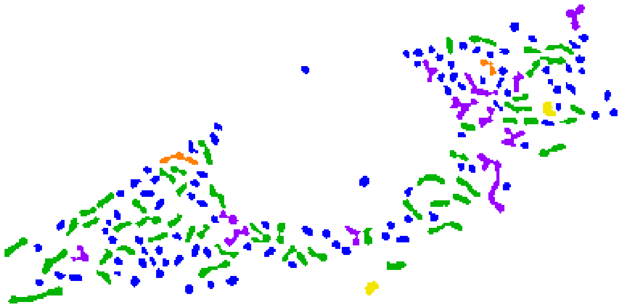

(c) Object classification image.

Figure S3: Image processing results of a cell from z-IETD treatment. See Figure S17 for mitochondria subtype color codes.

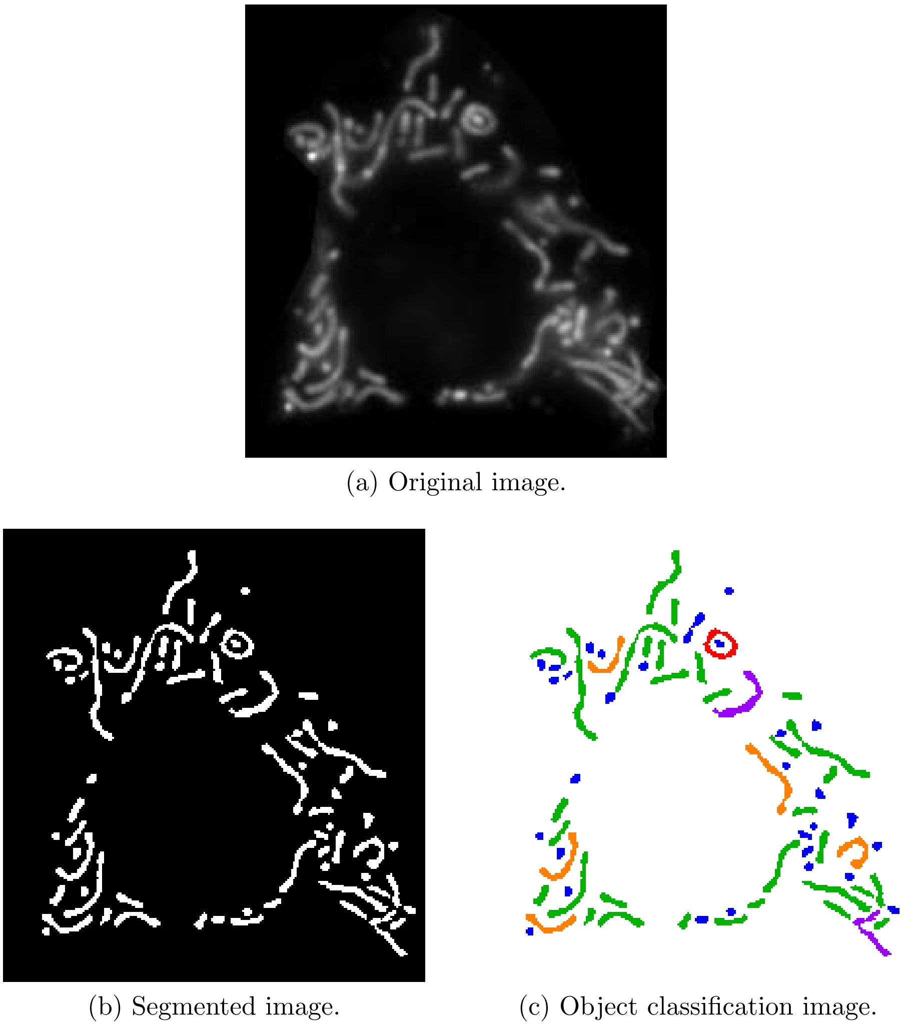

(a) Original image.

(b) Segmented image.

(c) Object classification image.

Figure S4: Image processing results of a cell from z-LEHD treatment. See Figure S17 for mitochondria subtype color codes.

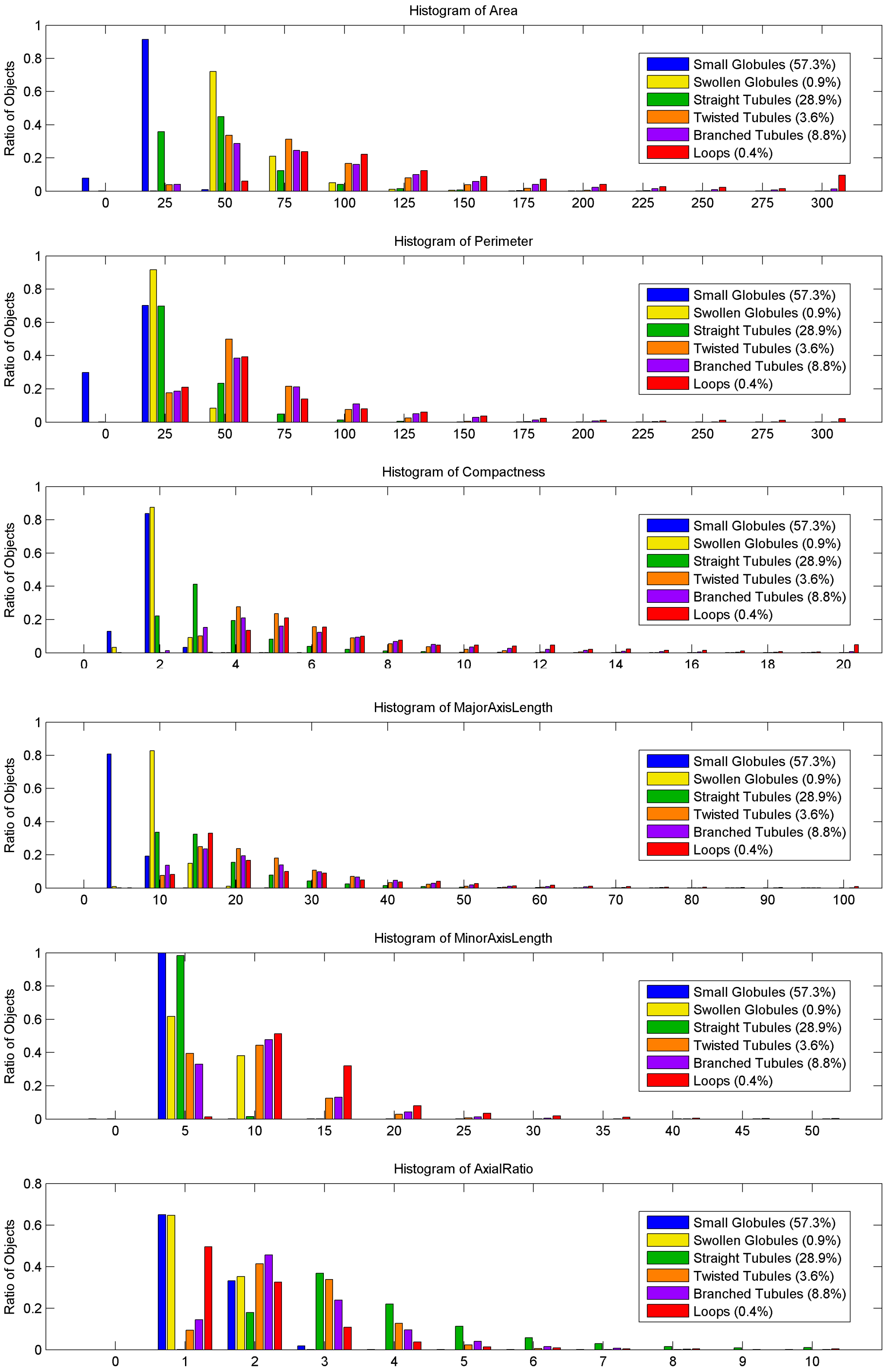

Figure S5: Histograms of morphology features for different mitochondrial subtypes.

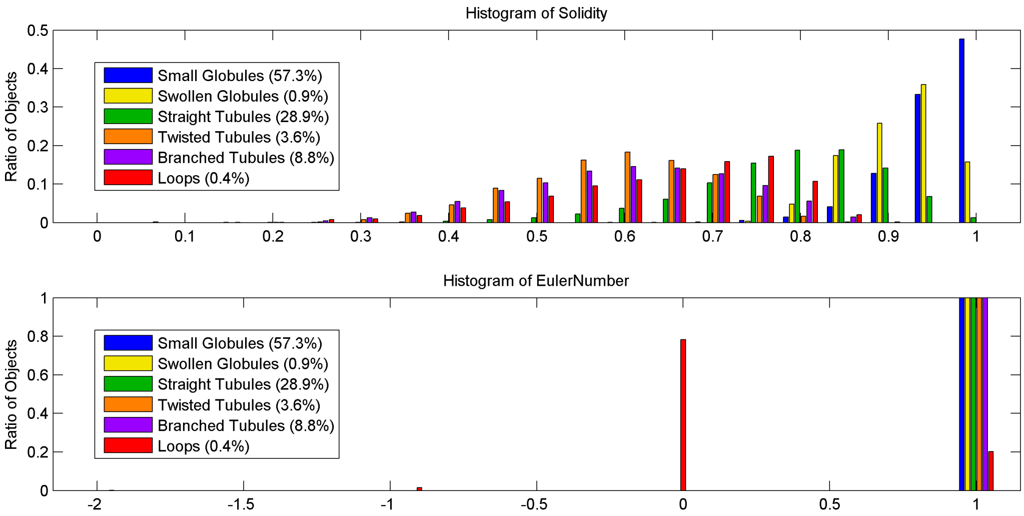

Figure S5: (Cont.) Histograms of morphology features for different mitochondrial subtypes.

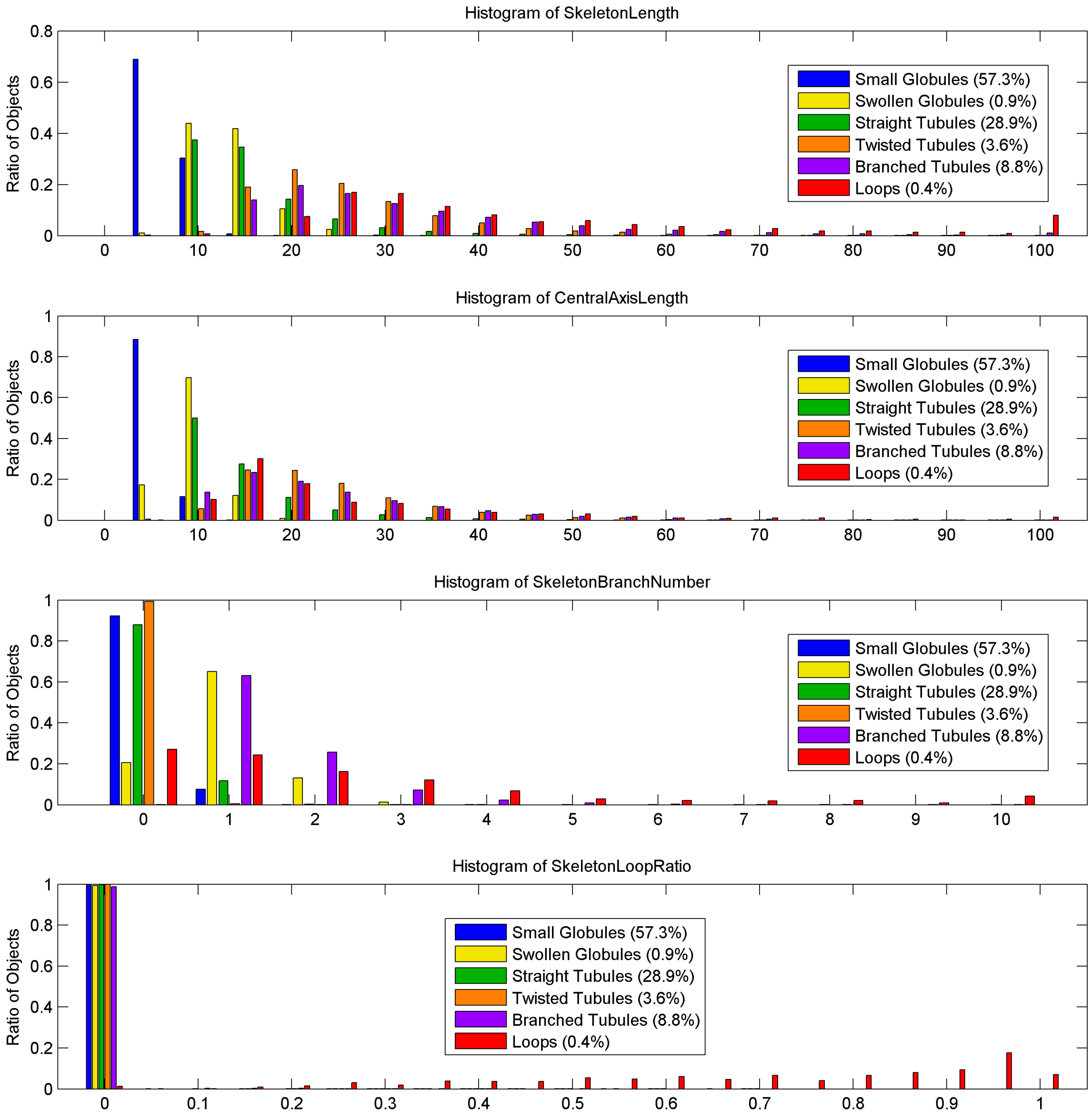

Figure S6: Histograms of skeleton features for different mitochondrial subtypes.

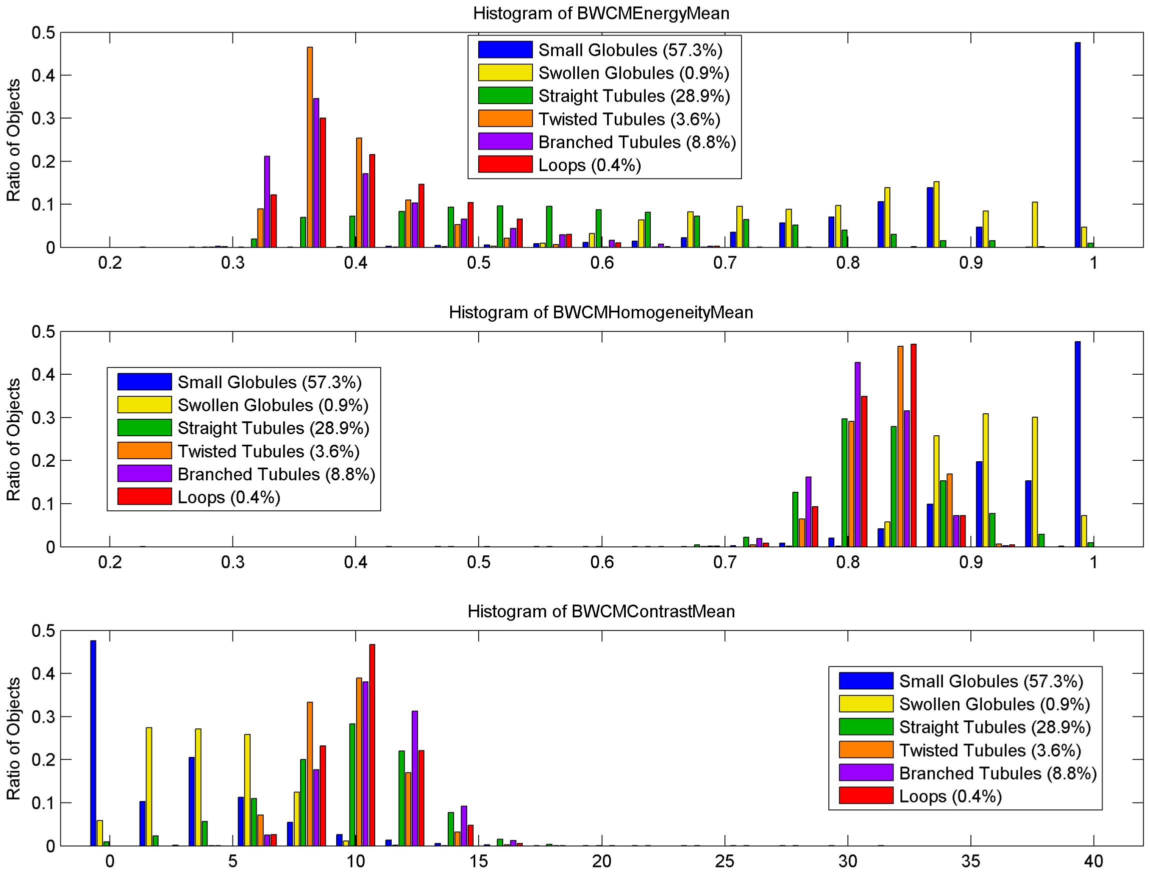

Figure S7: Histograms of binary texture features for different mitochondrial subtypes.

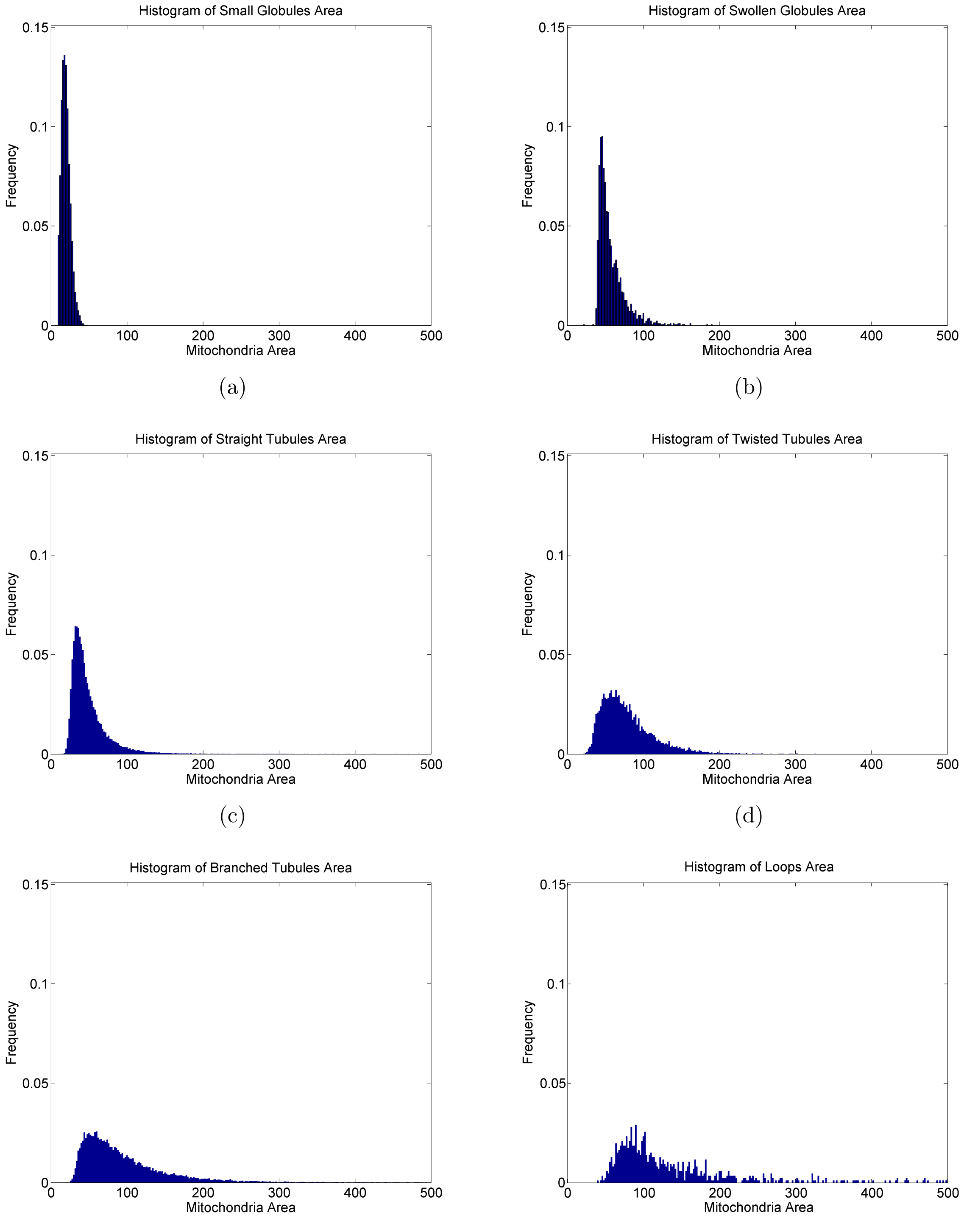

Figure S8: Area distributions of mitochondria.

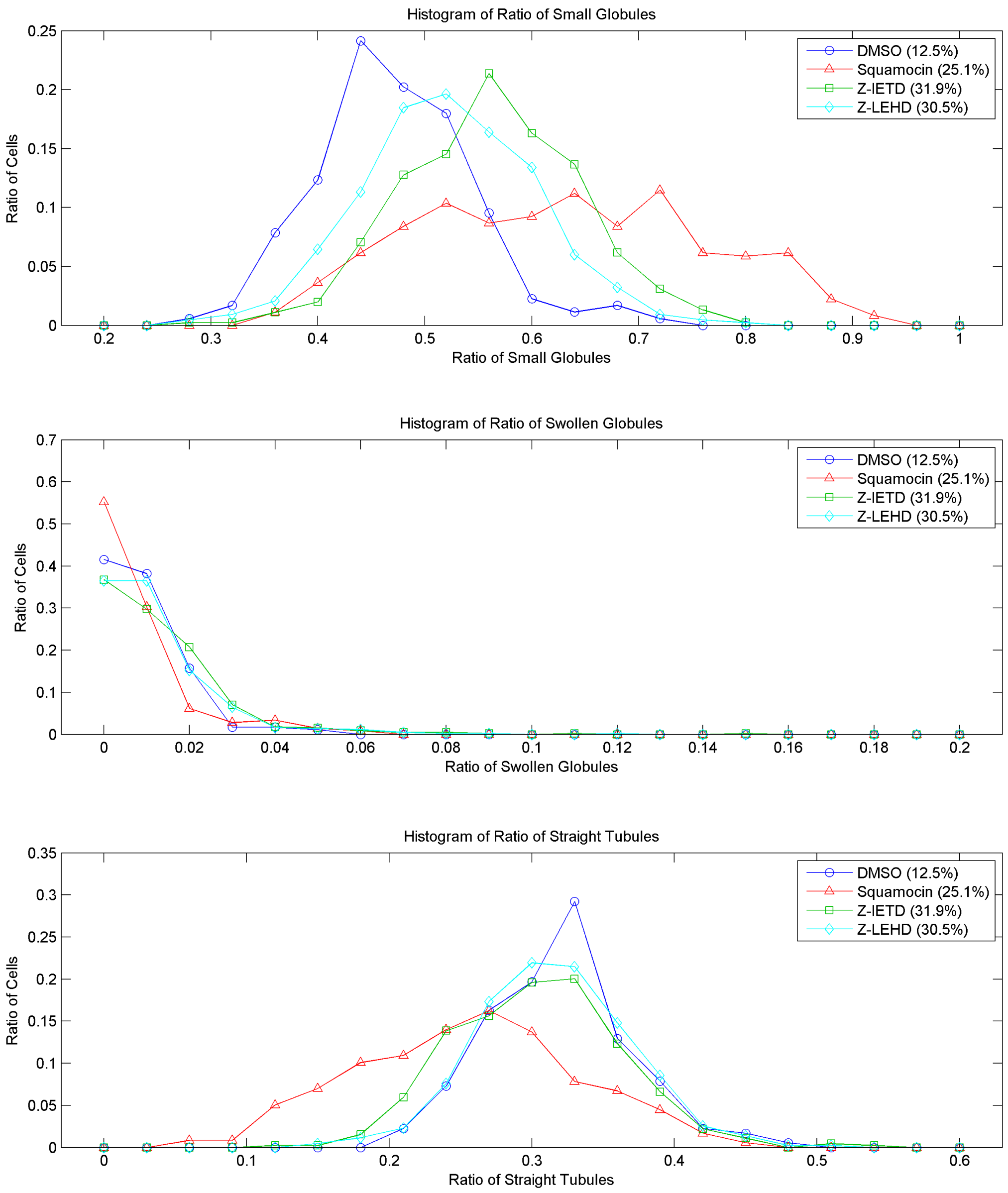

Figure S9: Histogram of mitochondria subtype ratios in cells separated by treatment.

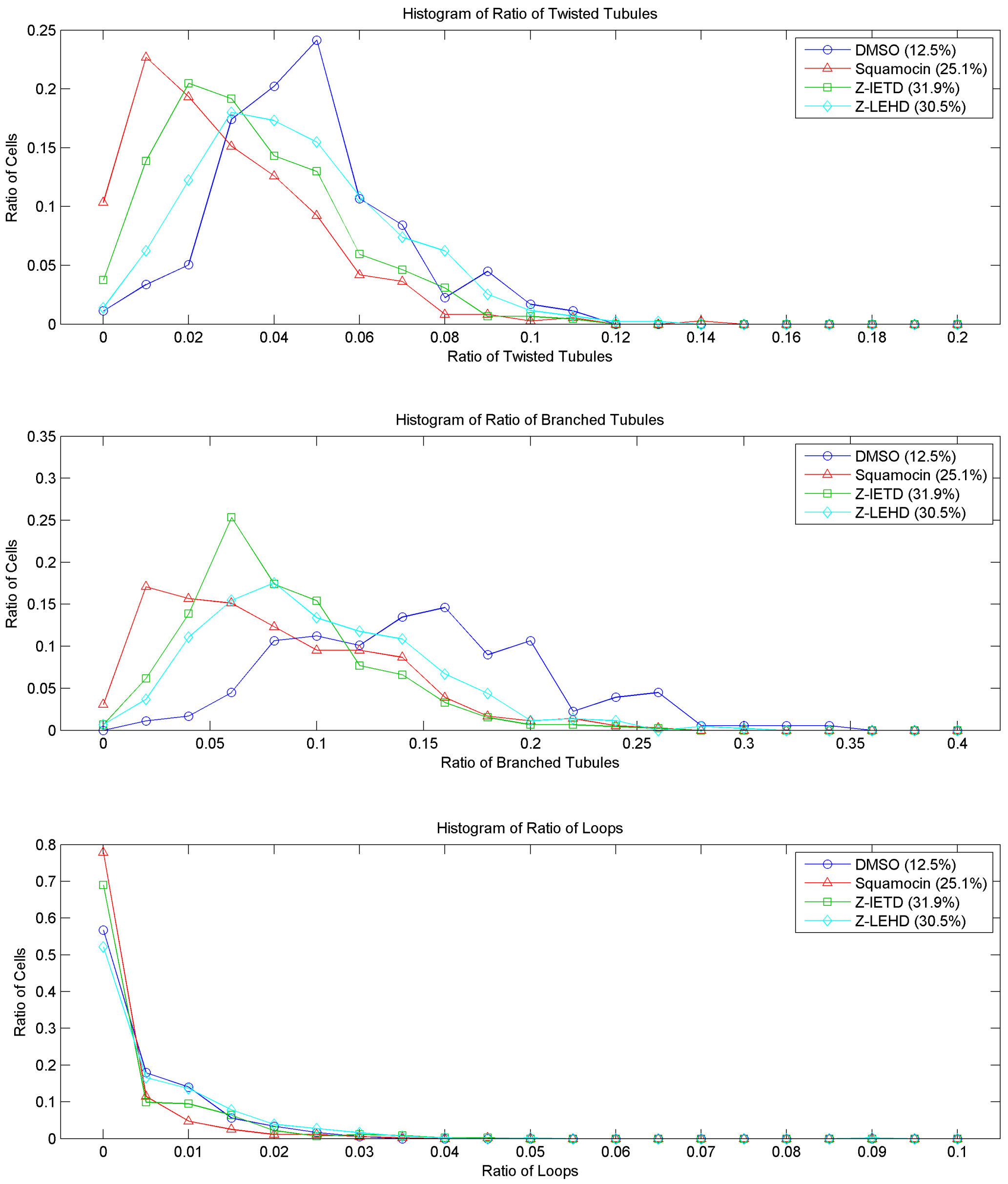

Figure S9: (Cont.) Histogram of mitochondria subtype ratios in cells separated by treatment.

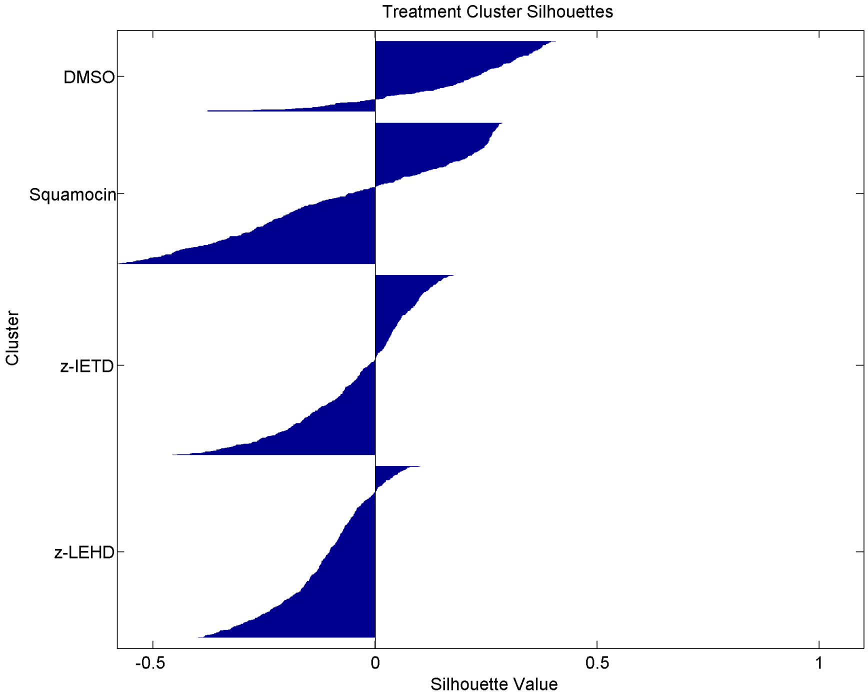

Figure S10: Silhouette graph for cell treatment clusters.

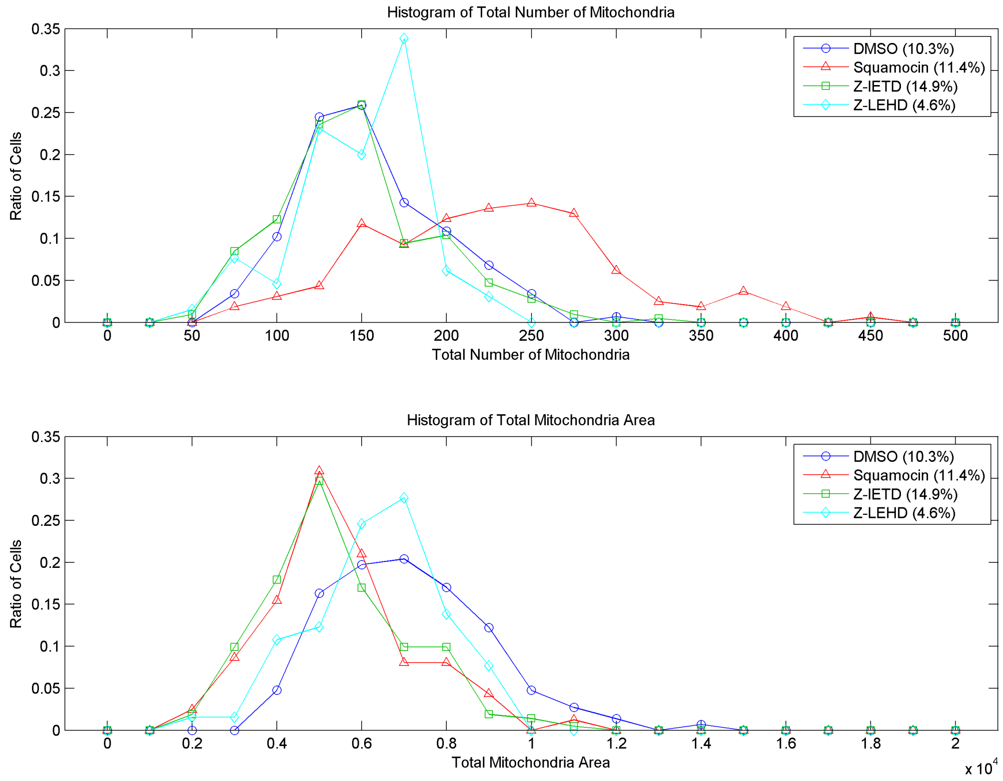

Figure S11: Distribution of the total number and area of mitochondria in only representative cells under different treatments.

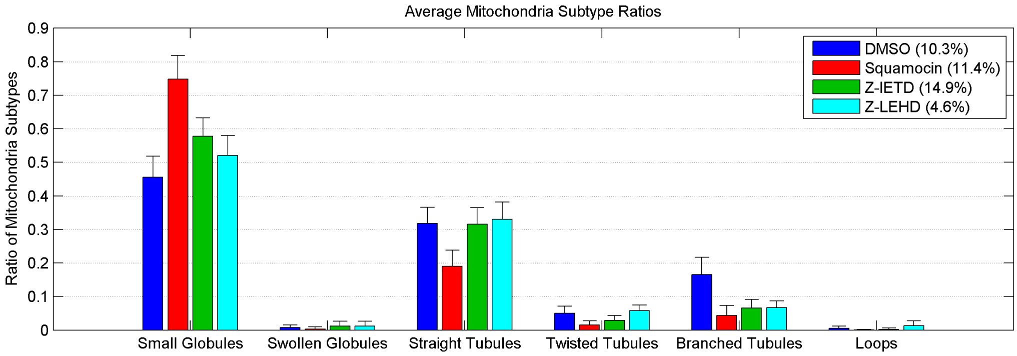

Figure S12: Average ratio of mitochondrial subtypes in only representative cells given different treatments.

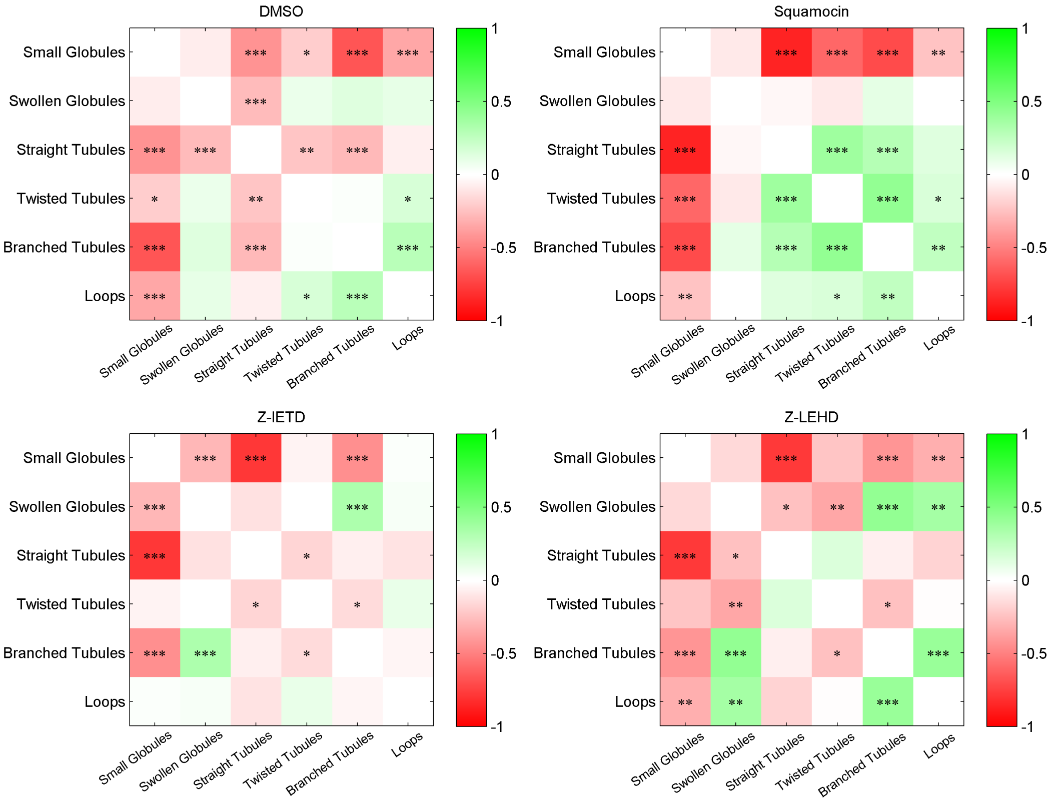

Figure S13: Correlation heat map of the ratios of all pairs of subtypes in only representative cells of different treatments. For each pair, * indicates $p < 0.05$, ** indicates $p < 0.01$, and *** indicates $p < 0.001$.

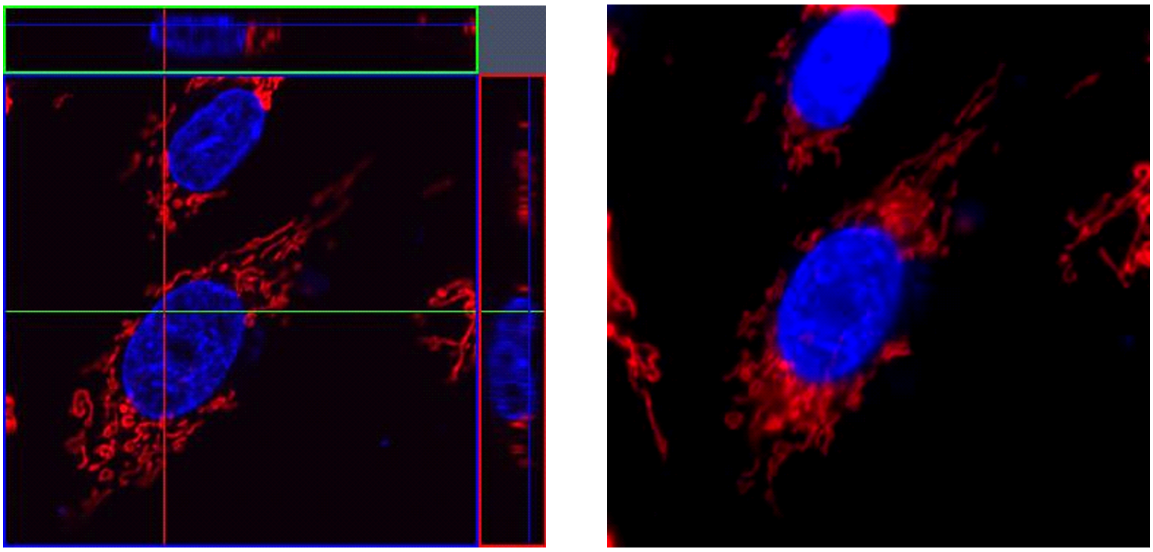

(a) 3D confocal fluorescence micrograph.

(b) 2D epi-fluorescence micrograph.

Figure S14: 2D (epi-fluorescence microscopy) and 3D (confocal fluorescence microscopy) micrographs of a control cell with tubular mitochondria. See text for details.

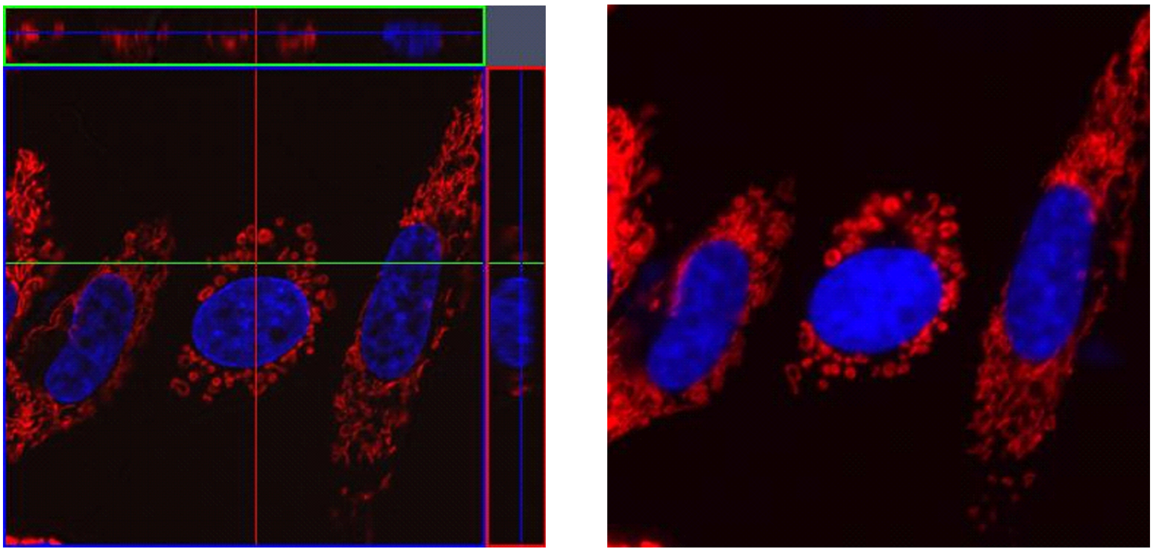

(a) 3D confocal fluorescence micrograph.

(b) 2D epi-fluorescence micrograph.

Figure S15: 2D (epi-fluorescence microscopy) and 3D (confocal fluorescence microscopy) micrographs of a squamocin-treated cell with mitochondrial donuts. See text for details.

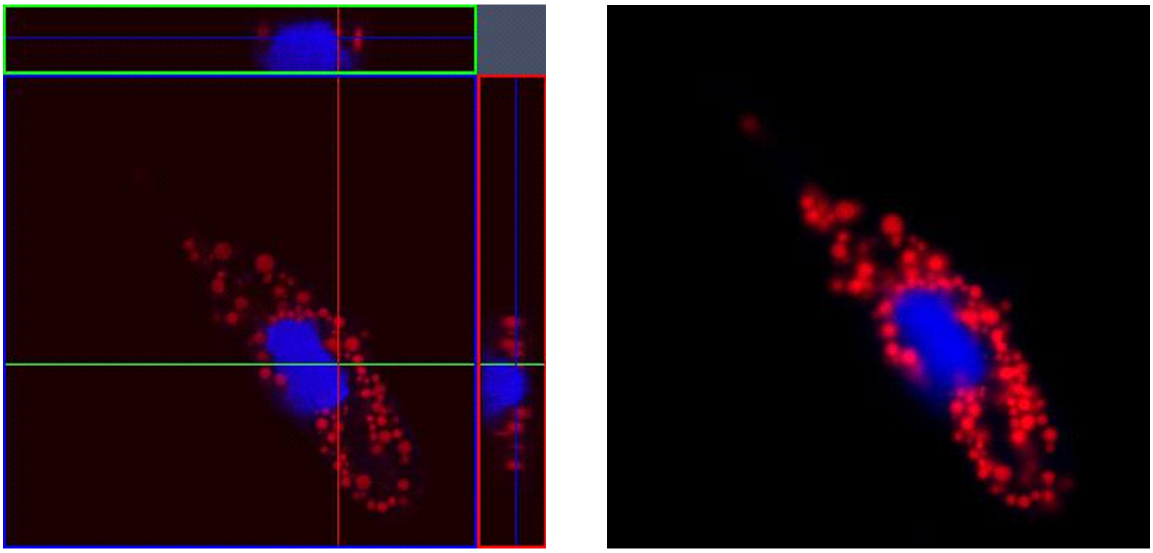

(a) 3D confocal fluorescence micrograph.

(b) 2D epi-fluorescence micrograph.

Figure S16: 2D (epi-fluorescence microscopy) and 3D (confocal fluorescence microscopy) micrographs of a squamocin-treated cell with swollen mitochondrial globules. See text for details.

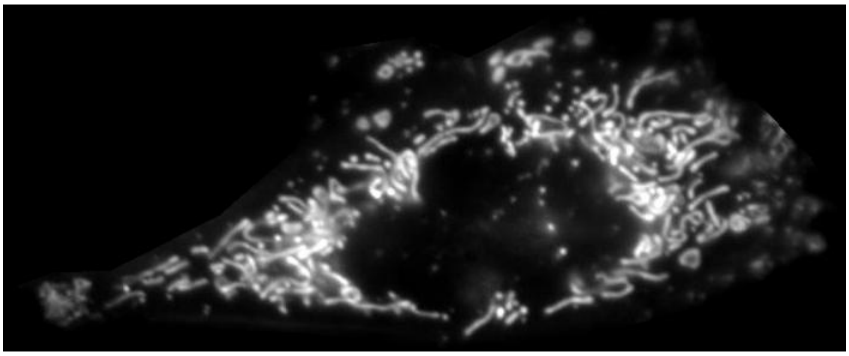

(a) Original image of fluorescent mitochondria from a single cell.

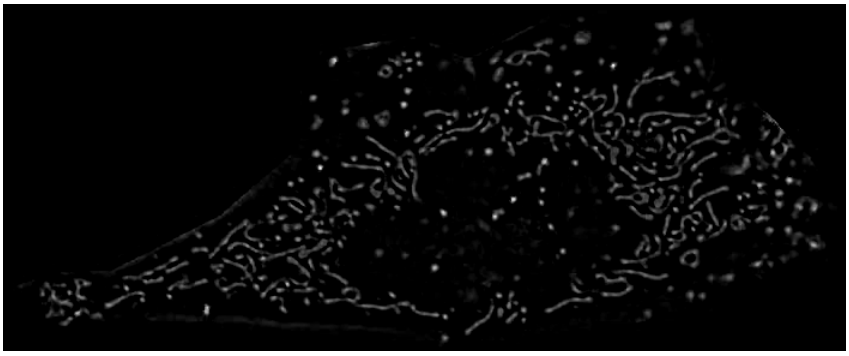

(b) Normalized image using adaptive local normalization.

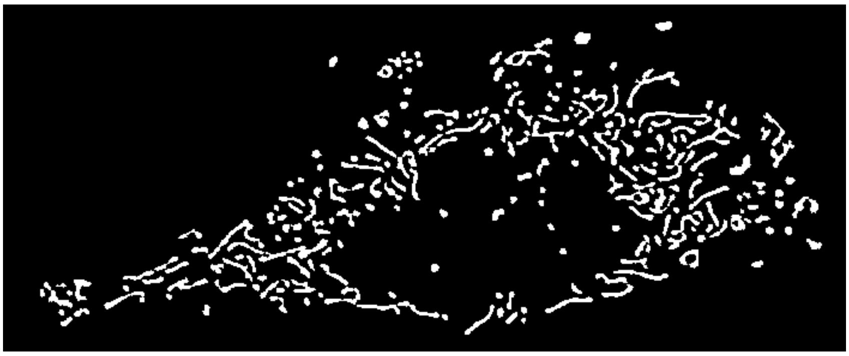

(c) Segmented image using Otsu's thresholding.

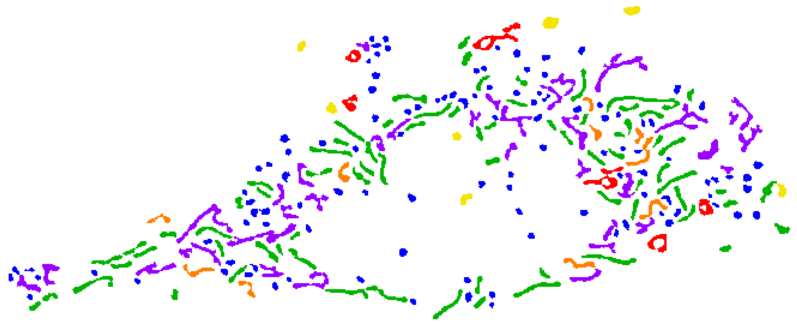

(d) Object classification by machine learning.

Figure S17: Image processing on a cell micrograph of fluorescent mitochondria. The original image (a) is adaptively normalized to enhance salient subcellular structures and remove background variation, then Otsu's thresholding is applied to the normalized image (b) to produce the final segmentation (c). The bottom image (d) shows morphological subtyping of mitochondria using machine learning, individual mitochondria is color-coded as follows: blue - small globules, yellow - swollen globules, green - linear tubules, orange - twisted tubules, purple - branched tubules, red - loops.

Morphological features	Area	a
	Perimeter	p
	Compactness	$p^2/(4\pi a)$
	Major axis length	l_{major}
	Minor axis length	l_{minor}
	Axial ratio	l_{major}/l_{minor}
	Solidity	a/a_{cv}
	Euler number	e
Skeleton features	Skeleton length	l_s
	Skeleton ratio	l_s^2/a
	Principle axis length	l_c
	Principle axis ratio	l_c^2/a
	Branch point number	b
	Loop ratio	r_{loop}
Binary texture features	BWCM Energy Mean	
	BWCM Energy Range	
	BWCM Homogeneity Mean	
	BWCM Homogeneity Range	
	BWCM Contrast Mean	
	BWCM Contrast Range	

Table S1: Mitochondrial features used for machine learning.

Total Statistics	Total Number of Mitochondria
	Total Mitochondria Area
	Total Mitochondria Length
Subtype Ratios	Ratio of Small Globules
	Ratio of Swollen Globules
	Ratio of Straight Tubules
	Ratio of Twisted Tubules
	Ratio of Branched Tubules
	Ratio of Loops
Area Ratios	Area Ratio of Small Globules
	Area Ratio of Swollen Globules
	Area Ratio of Straight Tubules
	Area Ratio of Twisted Tubules
	Area Ratio of Branched Tubules
	Area Ratio of Loops
Length Ratios	Length Ratio of Small Globules
	Length Ratio of Swollen Globules
	Length Ratio of Straight Tubules
	Length Ratio of Twisted Tubules
	Length Ratio of Branched Tubules
	Length Ratio of Loops

Table S2: Cell features used for profiling cell responses measured in terms of the composition of morphologial subtypes of mitochondria.
